# Genome–phenome wide association study of broadly defined headache

**DOI:** 10.1093/braincomms/fcad167

**Published:** 2023-05-24

**Authors:** Wan-Ting Hsu, Yu-Ting Lee, Jasmine Tan, Yung-Han Chang, Frank Qian, Kuei-Yu Liu, Jo-Ching Hsiung, Chia-Hung Yo, Sung-Chun Tang, Xia Jiang, Chien-Chang Lee

**Affiliations:** Department of Epidemiology, Harvard T.H. Chan School of Public Health, Boston, MA 02115, USA; Health Data Science Research Group, National Taiwan University Hospital, Taipei 100, Taiwan; Health Data Science Research Group, National Taiwan University Hospital, Taipei 100, Taiwan; Department of Emergency Medicine, National Taiwan University Hospital, Taipei 100, Taiwan; Department of Biostatistics, University of California, Los Angeles, CA 90095, USA; Department of Medicine, Beth Israel Deaconess Medical Center, Harvard Medical School, Boston, MA 02215, USA; Health Data Science Research Group, National Taiwan University Hospital, Taipei 100, Taiwan; Department of Pediatrics, Einstein Medical Center-Philadelphia, National Taiwan University Hospital, Philadelphia, PA 19141, USA; Department of Emergency Medicine, Far Eastern Memorial Hospital, New Taipei City 220, Taiwan; Department of Neurology, National Taiwan University Hospital, Taipei 100, Taiwan; Department of Epidemiology, Harvard T.H. Chan School of Public Health, Boston, MA 02115, USA; Department of Clinical Neuroscience, Center for Molecular Medicine, Karolinska Institutet, 171 76 Stockholm, Sweden; Health Data Science Research Group, National Taiwan University Hospital, Taipei 100, Taiwan; Department of Emergency Medicine, National Taiwan University Hospital, Taipei 100, Taiwan; The Center for Intelligent Healthcare, National Taiwan University Hospital, Taipei 100, Taiwan

**Keywords:** headache, migraine, genome-wide association study, phenome-wide association study, PheWAS

## Abstract

Until recently, most genetic studies of headache have been conducted on participants with European ancestry. We therefore conducted a large-scale genome-wide association study of self-reported headache in individuals of East Asian ancestry (specifically those who were identified as Han Chinese). In this study, 108 855 participants were enrolled, including 12 026 headache cases from the Taiwan Biobank. For broadly defined headache phenotype, we identified a locus on Chromosome 17, with the lead single-nucleotide polymorphism rs8072917 (odds ratio 1.08, *P* = 4.49 × 10^−8^), mapped to two protein-coding genes *RNF213* and *ENDOV*. For severe headache phenotype, we found a strong association on Chromosome 8, with the lead single-nucleotide polymorphism rs13272202 (odds ratio 1.30, *P* = 1.02 × 10^−9^), mapped to gene *RP11-1101K5.1*. We then conducted a conditional analysis and a statistical fine-mapping of the broadly defined headache-associated *loci* and identified a single credible set of *loci* with rs8072917 supporting that this lead variant was the true causal variant on *RNF213* gene region. *RNF213* replicated the result of previous studies and played important roles in the biological mechanism of broadly defined headache. On the basis of the previous results found in the Taiwan Biobank, we conducted phenome-wide association studies for the lead variants using data from the UK Biobank and found that the causal variant (single-nucleotide polymorphism rs8072917) was associated with muscle symptoms, cellulitis and abscess of face and neck, and cardiogenic shock. Our findings foster the genetic architecture of headache in individuals of East Asian ancestry. Our study can be replicated using genomic data linked to electronic health records from a variety of countries, therefore affecting a wide range of ethnicities globally. Our genome–phenome association study may facilitate the development of new genetic tests and novel drug mechanisms.

## Teaser

Hsu *et al*. conducted a genome-wide association study on headaches in East Asians, followed by a phenome-wide association analysis using the UK Biobank. Single-nucleotide polymorphism rs8072917 of gene RNF213 was the causal variant for broadly defined headaches, which was also associated with cellulitis, neck and face abscesses and muscular symptoms.

## Graphical Abstract

**Graphical Abstract:**
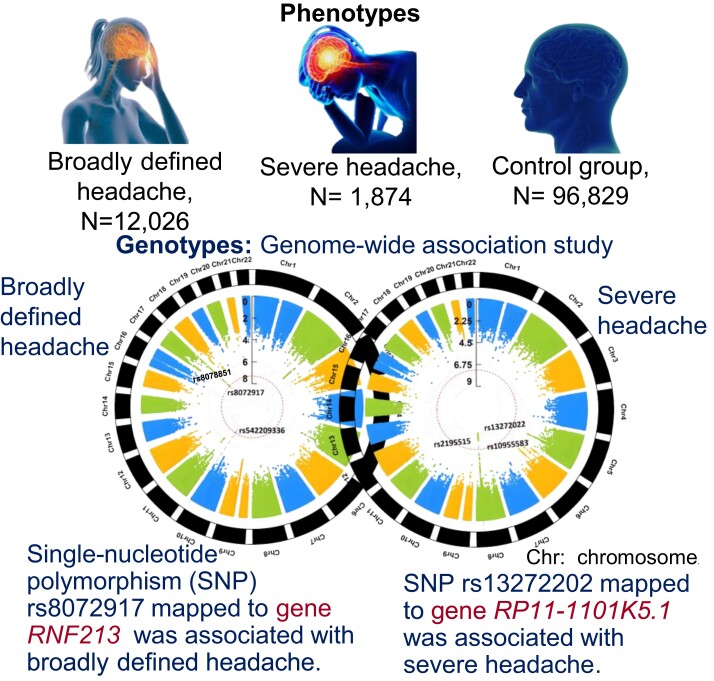

## Introduction

Headache has been recognized as one of the most common neurological disorders worldwide and has been classified into primary and secondary headaches.^1–3^ Primary headaches are tension-type, migraine, cluster, or other trigeminal autonomic cephalalgia headaches.^3^ Secondary headaches are defined as any headache caused by another condition, such as infections, space-occupying lesions, vascular abnormalities, metabolic disorders, or medications.^3^ In 2013, headache was ranked as the third most common global cause of disability based on years lived with disability (YLD).^1^ The reported annual incidence is about 14.2 cases of tension-type headache (TTH) per 1000 person-years and 8.1 cases of migraine per 1000 person-years.^4^ More specifically, migraine was the second leading cause of YLD among young adults and middle-aged women worldwide.^5^ While migraine prevalence varies across ethnicities, migraine is the most common cause of disability among other neurological disorders reported worldwide.^5^ In previous epidemiological studies,^6,7^ Asians were found to have a lower migraine prevalence than Western populations, but the mechanism underlying the ethnic diversity has yet to be properly elucidated. Furthermore, migraine contributed to an estimated economic burden of up to 17 billion dollars in direct and indirect costs in the USA yearly.^8^ In Europe, the total annual cost for the management of headache was 111 billion euros among adults with migraines and 21 billion euros among those with TTH.^9^

For most of the classification systems to date, the differential diagnosis between migraine and TTH mostly depends on self-reported associated features. However, distinguishing a TTH attack from a mild to moderate migraine headache, the most common episodic headache attack, has long been recognized as a diagnostic challenge.^10^ According to the ICHD-3 criteria, defining TTH or migraine depends on the presence (migraine) or the absence (TTH) of specific features, such as unilateral location, pulsating quality, moderate or severe pain intensity, photophobia and phonophobia, nausea and/or vomiting and aggravation by routine activity.^11^ The lack of distinctive positive features of TTH may contribute to its misdiagnosis as migraine. Moreover, previous epidemiological studies showed co-occurrence of migraine and TTH.^12^ Poor description of associated symptoms or the concomitant presentation of specific features of more than one headache type may complicate the diagnosis.

Alternatively, the continuum concept has been proposed and held that TTH and migraine may represent conditions along the same spectrum.^13^ Many migraine attacks are accompanied by prototypical TTH-like symptoms such as muscle tension and neck pain.^10^ Conversely, those with TTH are often accompanied by migraine-like symptoms such as photophobia, phonophobia and aggravation by activity^14^ These findings are in support of a continuum perspective and emphasize the potential role of shared pathophysiologic mechanisms.

Studies of families and twins have provided evidence for a genetic component in both migraine and TTH, with a heritability of ∼40–50%^15,16^ Previous genome-wide association studies (GWAS) have identified many genetic *loci* associated with migraine, and genome-wide meta-analysis of Gormley *et al.*^17^ has identified 38 genetic susceptibility *loci*. No GWAS to date have been conducted for TTHs. A recent GWAS of Meng *et al.*^18^ from UK Biobank (UKB) identified thousands of single-nucleotide polymorphisms (SNPs) representing 28 genomic *loci* associated with a ‘broadly defined’ headache phenotype, instead of any specific type of headache. Of note, half of these *loci* were represent novel associations not previously shown in the migraine genome-wide meta-analyses. Since only two (rs11172113 and rs9349379) of 28 genomic loci were shown in migraine genome-wide meta-analysis Gormley *et al.*^17^, the GWAS using broadly defined headaches in the UKB identified novel associations. More comprehensively, a recent joint GWAS meta-analysis of these two cohorts (self-reported migraine cohort by Gormley *et al*. and self-reported headache cohort by Meng *et al*.) identified another four new genetic loci, which demonstrated that the genetically correlated phenotypes could be reasonably meta-analysed together in practice to identify additional genetic components.^19^ These findings suggest that the genetic aetiology of headache can involve additional unidentified mechanisms, and using broadly defined headaches as an endpoint may boost study power and help us discover potentially novel genetic variants and pathways.

Genetic studies conducted to date have largely been limited to participants of European ancestry. However, common gene variants associated with headache in East Asian ancestry (specifically those who identify as Han Chinese) remain not well understood. To our knowledge, there have been no GWAS of broadly defined headaches in the Han Chinese population. The discovery of genetic variants associated with headache may help to improve the understanding of underlying biological mechanisms and facilitate the discovery of novel management strategies. To provide new insights into the genetic architecture of broadly defined headaches, we conducted a large GWAS of self-reported headaches in research participants from the Taiwan Biobank (TWB).

## Methods

### Study population

This study incorporated 114 089 Taiwanese Han Chinese subjects randomly sampled from the TWB. TWB is a healthcare research database that aims to investigate associations between genetics, life styles, drug use or progression of varied disease. TWB has finished recruiting and monitoring a cohort of 200 000 healthy individuals from the general Taiwanese population without a history of cancer and another cohort of 100 000 patients with chronic diseases.^20^ Inclusion criteria were Taiwanese Han Chinese individuals aged between 30 and 70 years old whose ancestors were self-reported as Taiwanese Han Chinese. To avoid confounding from population stratification, aboriginal people and individuals of non-East Asian ancestry were also excluded. Additionally, we excluded kinship (i.e. ancestry of Taiwanese Han Chinese only one generation back) as well as self-reported schizophrenia, depression, manic depression, postpartum depression, obsessive-compulsive disorder, alcoholism and drug abuse. Participants completed a detailed clinical, demographic and lifestyle questionnaire, underwent clinical measures, provided biological samples (blood, urine and saliva) for future analysis and agreed to have their health records accessed. TWB database is managed and regulated by the Ministry of Health and Welfare in Taiwan and is accessible to all the researchers in Taiwan to allow studies focusing on the relationship between genetics, environment, diet and the causes and prognosis of diseases. Informed consent was obtained from all participants prior to their enrolments in TWB research. Details of the TWB resource can be found at the website of TWB.^21^

### Ethic approval and patient consent

This research project was approved by the ethics committee of National Taiwan University Hospital Institutional Review Board. The study was conducted in accordance with the principles of the Declaration of Helsinki and the Good Clinical Practice Guidelines, and all the participants were informed consent.

### Study variable

To assess the impact of headache, numerous broadly defined or migraine-specific patient-reported outcome measures (PROMs) have been established in previous studies.^22^ These include six-item Headache Impact Test (HIT-6), Migraine-Specific Questionnaire (MSQ), Migraine Disability Assessment (MIDAS) questionnaire and Migraine Treatment Optimization Questionnaire (M-TOQ), of which some have demonstrated strong evidence of content validity supportive for use in clinical practice.^23^ Based on a similar approach, the Academia Sinica, Taiwan developed a questionnaire with a broad range of patient-reported measures, including ratings of headache severity, headache-related disability and migraine-related symptoms.

Participants of TWB were asked to fill a detailed pain-related questionnaire at baseline on demographics, previous medical history including headache or migraine and other types of pain (joint pain and stiffness all over the body, neck or shoulder pain, low back pain, sciatic nerve pain and dysmenorrhoea for female participants who did not have menopause over the last 3 months) between December 2008 and November 2020 (Supplementary Table 5). Participants could select more than one option. The questionnaire asked participants about the frequency (constant, come and go or prefer not to say) of their headaches or migraines and pains and severity (mild, moderate or severe) of their headaches or migraines over the last 3 months. They answered ‘yes’ or ‘no’ if they had any of the following symptoms with their headaches: nausea, vomiting and light sensitivity. The participants further answered if their headaches affected their work or study (yes or no). They were also asked about their family history of headaches and migraines. The definition of broadly defined headache phenotype was used as a case in our study. Participants with broadly defined headache reported nausea or vomiting affecting their work or study and were assigned to the cases of severe headache phenotype. Participants without headache and migraine, and without a family history of headaches and migraines, served as controls. We compared clinical characteristics of broadly defined headache, severe headache and no headache using chi-squared test for categorical variables and one-way ANOVA for continuous variables.

### Genotyping and quality control

**Figure 1:**
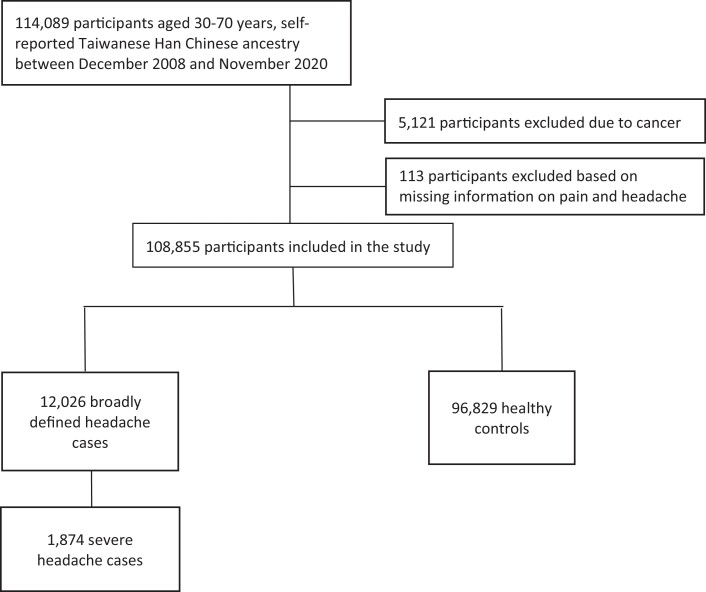
A flowchart showing the enrolment of headache cases and healthy controls using the TWB.

The customized Axiom-Taiwan Biobank Array Plate (TWB chip; Affymetrix Inc, CA, USA) was used to perform whole genome genotyping. In this study, we used the imputed genotype dataset. The TWBv2 array utilized whole-genome sequencing data from TWB participants to choose SNPs optimized for imputation in Han Chinese samples containing 660 606 marker SNPs and the imputed genotype dataset includes 9 809 486 marker SNPs. The ethics and governance council of TWB has released details on the genotype information and linkage disequilibrium (LD) of healthy people.^21^ We used PLINK to perform quality control procedures including sample quality, gender concordance and kinship for every individual.^24^ Potentially contaminated samples could be identified using a relatedness check. In PLINK, the command ‘–genome’ calculated identity-by-state (IBS) distance between pairwise samples using pairwise identity by descent (IBD). Pedigree errors, swaps, duplications, contamination and unknown family relationships were detected using IBD. Uncertain kinships were detected using PLINK 1.9 (https://zzz.bwh.harvard.edu/plink/ibdibs.shtml). To evaluate potential population stratification in our study, a principal component analysis was conducted. No outliers were identified from the scatter plot. Consequently, a total of 108 855 subjects, including 12 026 headache patients and 96 829 healthy controls, were retained for further analyses (Fig. 1). Quality control was also conducted for SNPs. SNPs failed Hardy–Weinberg tests with *P* < 10^−6^, genotype missing rate > 5% and minor allele frequency (MAF) < 0.5% were eliminated. As a result, we included 4 230 987 SNPs for the analysis.

### Statistical analysis

In our study, PLINK was used as the main GWAS analysis software. We removed SNPs with INFO scores < 0.8, with MAF < 0.5% or those that failed Hardy–Weinberg tests *P* < 10^−6^. SNPs on the X and Y chromosomes and mitochondrial SNPs were excluded from analyses.

### GWAS analysis

Association testing was carried out using multivariate logistic regression implemented in PLINK, adjusting for age, sex and the first five population principal components to control for population stratification. The SNPs used for principal components were randomly selected from the pool of autosomal SNPs on TWB 2.0 array with the following criteria: MAF > 5%, low inter-marker LD (*r*^2^ < 0.3), call-rate >99% and Hardy–Weinberg equilibrium (*P* > 10^−4^). Odds ratios (ORs) were calculated by considering the non-risk allele as a reference. The minimum *P*-value was determined under three genetic models (additive, recessive and dominant). The genomic inflation factor was derived by applying *P*-values from logistic regression in an additive model for all the tested SNPs. SNP associations were considered significant if they had a *P* < 5 × 10^−8^. Manhattan plots and the corresponding quantile–quantile (Q–Q) plots were generated by PLINK and were used to examine the *P*-value distribution. For general statistical analysis, we used R statistical environment version 3.51 or PLINK version 1.9.^24^

### LD score regression

We used LD score regression (LDSC) to examine SNP-based heritability for broadly defined headache and to assess whether there was population stratification.^25^ Pre-computed LD scores for East Asian populations based on 1000 Genomes Project data were used as a reference panel.

### Gene-based analysis

Gene-based genome-wide association analysis was performed with MAGMA v1.6, which was integrated in FUMA.^26^ Summary statistics of SNPs are aggregated to the level of whole genes, testing the joint association of all SNPs in the gene with the phenotype. Briefly, variants were assigned to protein-coding genes (*n* = 18 297; Ensembl build 85) if they are located in the gene body, and the resulting SNP *P*-values are combined into a gene test statistic using the SNP-wise mean model. According to the number of tested genes, the level of genome-wide significance was set at 0.05/17 311 = 2.9 × 10^−6^.

### Functional mapping

To interpret GWAS results, functional annotation was performed through SNP2GENE process in the FUMA platform, in which SNPs were annotated with their biological functionality and mapped to genes based on positional, eQTL and chromatin interaction information of SNPs.^27^

First, independent significant SNPs in the GWAS summary statistics and their surrounding genomic *loci* were identified based on their *P*-values (*P* < 5 × 10^−8^) and independence from each other (*r*^2^ < 0.6 in the 1000G phase 3 reference panel) within a 1-Mb window. Thereafter, lead SNPs were identified from independent significant SNPs and defined as SNPs independent of each other at *r*^2^ < 0.1. For further annotation, candidate SNPs were identified and defined as SNPs that were in LD with the identified independent SNPs (*r*^2^ ≥ 0.6) within a 1-Mb window and were filtered with a MAF of ≥0.5% and GWAS *P*-value of >0.05. Thus, these SNPs may include SNPs that were not available in the GWAS input but are available in the 1000G reference panel and are in LD with an independently significant SNP.

Next, candidate SNPs together with independent significant SNPs were annotated in genomic risk *loci* based on several filters, including annotate variation (ANNOVAR) for functional consequences on genes, CADD score (Combined Annotation-Dependent Depletion score, a score showing how deleterious the SNP is to protein structure or function, where 12.37 is the threshold to indicate potential pathogenicity), RegulomeDB scores (a categorical score ranging from 1 to 7 and representing regulatory functionality of SNPs, where lower score indicates greater evidence for having regulatory function), 15 chromatin states (127 tissues/cell types) from the Roadmap Epigenomics Project, eQTL data (GTEx v6 and v7), blood eQTL browser, BIOS QTL browser, BRAINEAC, MuTHER, xQTLServer, CommonMind Consortium and 3D chromatin interactions from HI-C experiments of 21 tissues/cell types.^27^ All filters above applied to selected SNPs were used to prioritize genes and thus influenced the number of SNPs mapped to genes.

Finally, genes were mapped using positional mapping, eQTL mapping and chromatin interaction mapping. Positional mapping is based on ANNOVAR annotations by specifying the maximum distance (default 10 kb) between SNPs and genes. eQTL mapping were tissue-specific and mapped SNPs to genes that likely affect expression of those genes up to 1 Mb (*cis*-eQTL). Chromatin interaction mapping was performed with significant chromatin interactions [defined as false discovery rate (FDR) < 1 × 10^−6^]. The two ends of significant chromatin interactions were defined as follows: Region 1, a region overlapping with one of the candidate SNPs; and Region 2, another end of the significant interaction, used to map to genes based on overlap with a promoter region (250-bp upstream and 50-bp downstream of the transcription start site by default).

### Gene-set analysis

Gene-set analysis was performed with MAGMA v1.6, which was integrated in FUMA.^26^ Briefly, results from the gene-based analyses (gene-level *P*-value) were used to test for association in 10 894 gene sets obtained from Msigdb v5.2, which included 4728 curated gene sets (including canonical pathways) and 6166 gene ontology terms. The Bonferroni-corrected significance threshold accounts for the total number of statistical gene-set tests and was set to 0.05/10 894 gene sets = 4.59 × 10^−6^. In gene-set analysis, individual genes were aggregated to groups of genes sharing certain biological, functional or other characteristics.

### Tissue expression analysis

To test the positive relationship between gene expression in a specific tissue and genetic associations, MAGMA gene-property analysis was performed to identify tissue specificity of the phenotype. In brief, gene-property analysis was based on the regression model using the result of gene analysis (gene-based *P*-value) and the average expression of genes per tissue type as the covariates. Gene expression values are log2 transformed average reads per kilobase of transcript per million reads mapped per tissue type after winsorized at 50 based on GTEx RNA-seq data. For 30 general and 53 specific tissue types, we performed two tissue expression analyses, using a Bonferroni correction for each.

### Conditional association analysis

The conditional analysis was conducted by PLINK to identify causal variants among the multiple independent signals using summary data from results of our above analyses. We used the standard GWAS (*P* < 5 × 10^−8^) threshold to define the secondary variants that were conditionally independent from the lead variant. An SNP is considered to be in LD with the top SNP if the *R*^2^ is >0.8. LocusZoom was used to plot SNPs along the chromosome and their associated positions (−log_10_*P*).

### Statistical fine-mapping

To further prioritize putative causal variants, we adopted Iterative Bayesian Stepwise Selection to conduct a statistical fine-mapping analysis using susieR (https://github.com/stephenslab/susieR/blob/master/vignettes/finemapping_summary_statistics.Rmd).^28^ We calculated posterior inclusion probability (PIP) for each SNP. We estimated the number of causal variants in each locus and created credible sets with a 95% cumulative PIP for containing a causal variant for broadly defined headache and severe headache groups. The PIP and 95% credible sets for broadly defined headache and severe headache were plotted using Susie plot.

### Phenome-wide association study

Following the results of our previous GWAS and conditional analysis, we performed a phenome-wide association study (PheWAS) using PheWAS R Package (https://github.com/PheWAS/PheWAS), aiming to explore multiple phenotypes associated with genetic variants. We tested the association Hardy of the individual variants (single variant PheWAS) with 1693 phenotype codes (‘phecodes’) defined by aggregating related ICD codes and 83 continuous biomarkers and traits in participants of White British ancestry in UKB. Age, sex and the first five population principal components were adjusted as covariates to correct for multiple testing of phecodes and continuous variables, we used a FDR-adjusted significance as a *P*-value cut-off.

## Results

### Cohort characteristics

A total of 1 14 089 individuals were invited to participate in the TWB questionnaire. After quality control, which involved excluding those with cancer and those with missing information on pain and headache sections, we identified 12 026 broadly defined headache cases and 96 829 controls for the GWAS association analysis. Among 12 026 headache cases, 1874 participants who reported that their headache conditions with nausea or vomiting had affected their work or study were assigned to another group as a severe headache phenotype.

The demographics and clinical characteristics of these cases and controls were summarized in Table 1. No headache control group had the most males (38.18%), followed by broadly defined headache (23.33%) and severe headache (13.67%). Of the three groups, the control group had the highest mean age (50.20 years), weight (64.07 kg) and height (162.11 cm). The control group also had the highest diabetes rates (5.91%), followed by broadly defined headache (4.97%) and severe headache (4.38%). Severe headache had a higher burden of chronic pulmonary disease (2.61%) and allergic disease (15.05%) across three groups.

**Table 1:** Characteristics of cohort of research participants from TWB reporting headache severity

Responders *N* = 108 855	Broadly defined headache *n* = 12 026	Severe headache *n* = 1874	No headache *n* = 96 829	*P*-values, comparisons
Male (%)	2806 (23.33%)	256 (13.67%)	36 974 (38.18%)	<0.001
Age (mean ± SD); years	47.14 ± 10.24	44.91 ± 9.61	50.20 ± 10.99	<0.001
Body weight (mean ± SD); kg	61.87 ± 11.99	60.03 ± 10.98	64.07 ± 12.80	<0.001
Body height (mean ± SD); cm	160.73 ± 7.70	159.93 ± 7.05	162.11 ± 8.37	<0.001
Systolic blood pressure (mean ± SD); mm Hg	115.68 ± 17.54	112.71 ± 16.33	121.02 ± 18.71	<0.001
Coronary heart disease	1.45%, 179	1.33%, 25	1.55%, 1497	0.747
Chronic pulmonary disease	2.10%, 252	2.61%, 49	1.18%, 1144	<0.001
Diabetes	4.97%, 598	4.38%, 82	5.91%, 5719	<0.001
Allergic disease	13.01%, 1564	15.05%, 282	9.39%, 9095	<0.001

### GWAS results

Association analysis was performed for 4 230 987 SNPs using logistic regression analysis on the basis of additive models after adjustment of age, sex and the first five population principal components.

**Figure 2:**
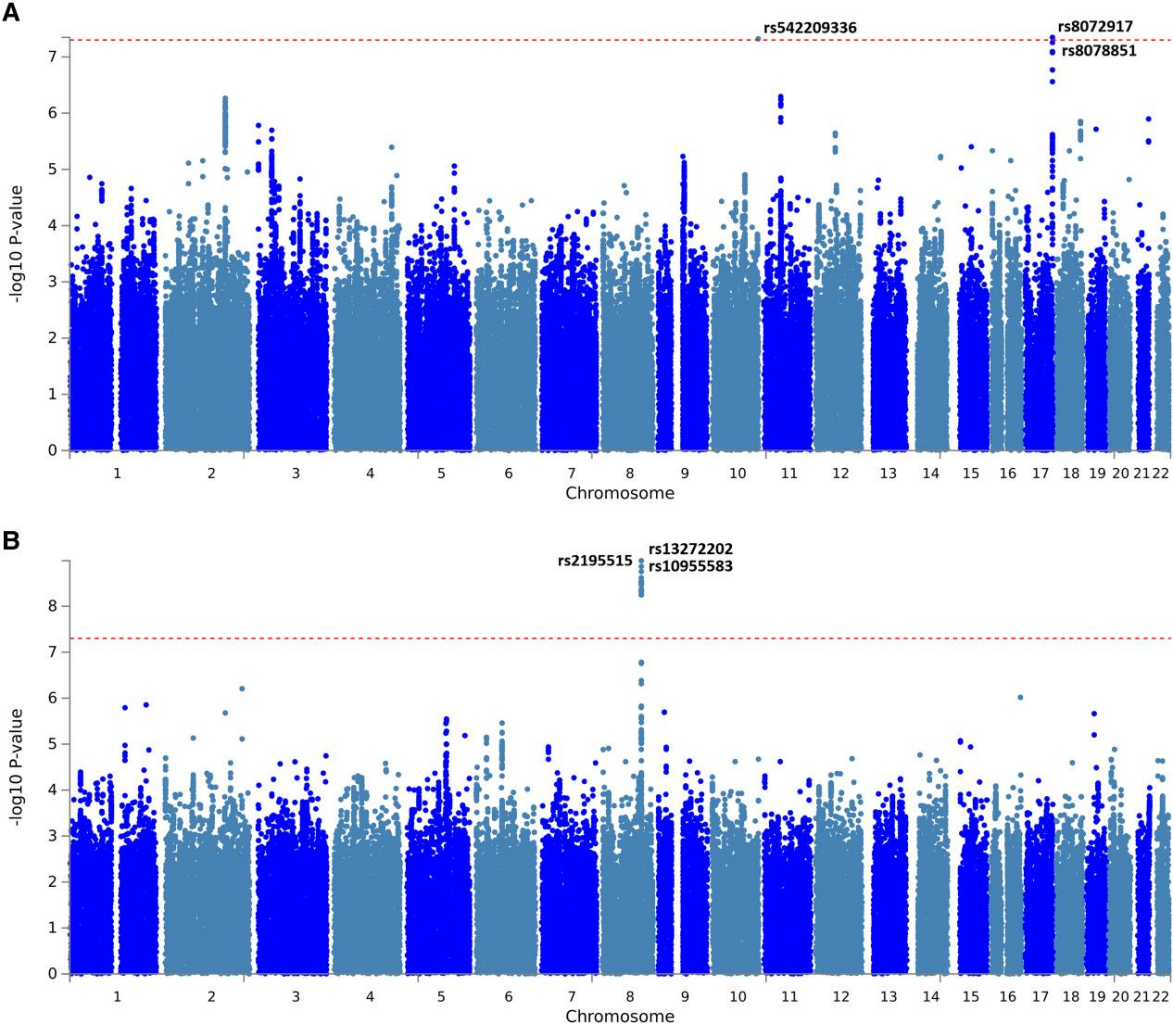
**Manhattan plots of the genetic associations with broadly defined headache (A) and severe headache (B) using the TWB cohort.** Association testing was carried out using multivariate logistic regression implemented in PLINK, adjusting for age, sex and the first five population principal components to control for population stratification. The *x*-axis shows the chromosomal positions, and the *y*-axis shows −log10 transformed *P*-values. The line indicates the genome-wide significance threshold (*P* < 5 × 10^−8^). (**A**) Three SNPs were identified as significantly associated with broadly defined headache: rs542209336 (*P* = 4.76 × 10^−8^) on Chromosome 10, rs8072917 (*P* = 4.49 × 10^−8^) and rs8078851 (*P* = 5.54 × 10^−8^) on Chromosome 17. (**B**) Significant associations with severe headache were observed on Chromosome 8, with SNP rs2195515 (*P* = 1.36 × 10^−9^), SNP rs13272202 (*P* = 1.02 × 10^−9^) and SNP rs10955583 (*P* = 1.76 × 10^−9^) being identified.

**Figure 3:**
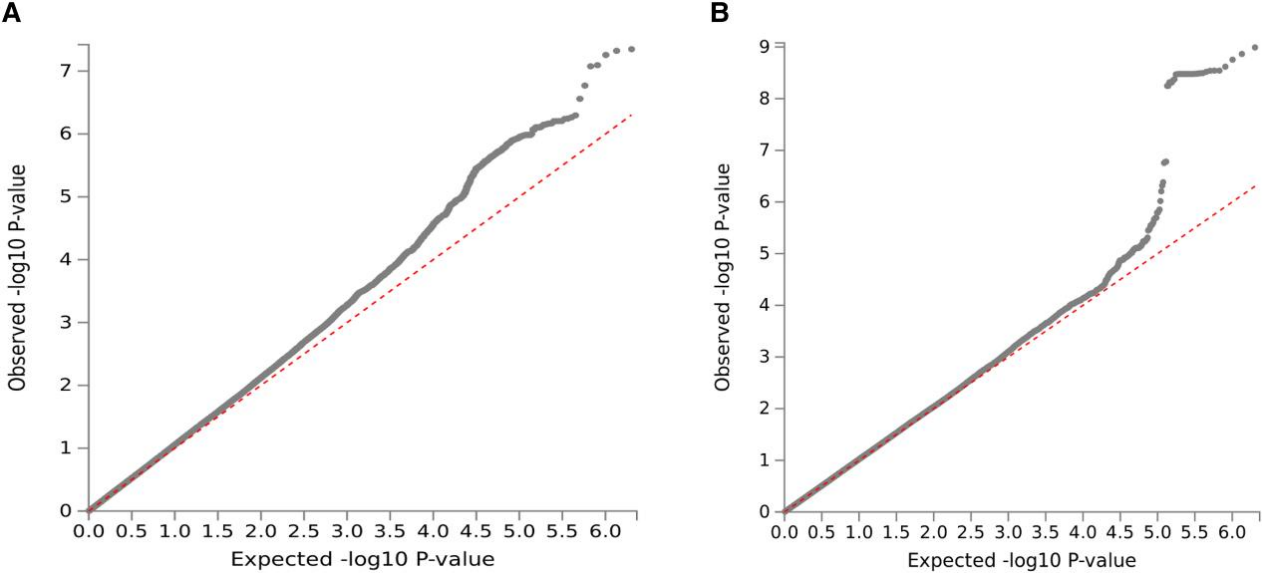
**Q–Q plots of GWAS on self-reported headache cases for broadly defined headache (A) and severe headache (B) using the TWB cohort.** On the Q–Q plots, the *x*-axis represents the −log10 of the expected *P*-values of the association from the chi-squared distribution, and the *y*-axis represents the −log10 of the observed *P*-values from the observed chi-squared distribution. Observationally, the grey dots depict the observed data with the top hit SNP coloured. The red line represents the expectation assuming there is no association under the null hypothesis. Grey lines and dots in the broadly defined headache plot deviate from 2.0, while grey lines and dots in the severe headache plot deviate from 4.0. Multiple genes might be involved in severe headache phenotype due to the larger deviations in the severe headache plot.

Figure 2 shows the Manhattan plots, and Fig. 3 depicts the Q–Q plot for all SNPs that passed quality control. When comparing Q–Q plots derived from different phenotypes, there was a slight difference in the upper right tail, indicating that severe headache phenotype might be affected by multiple different genes. For broadly defined headache phenotype, we identified an association that reached genome-wide significance (*P* < 5 × 10^−8^) on Chromosome 17, with lead SNP rs8072917 (OR 1.08, *P* = 4.49 × 10^−8^). For severe headache phenotype, we identified an association that reached genome-wide significance (*P* < 5 × 10^−8^) on Chromosome 8, with lead SNP rs13272202 (OR 1.30, *P* = 1.02 × 10^−9^) and 31 independent SNPs. Summary statistics for other genomic risk *loci* with *P* < 10^−6^ are available in Supplementary Table 1. Additionally, we conducted LDSC as a sensitivity analysis. The genomic inflation factor (*λ*GC) appeared at 1.0315. The LDSC intercept was 0.9992, which, being close to 1.0, indicated that most of the genome-wide elevation of the association statistics comes from true additive polygenic effects rather than residual population structure.

**Figure 4:**
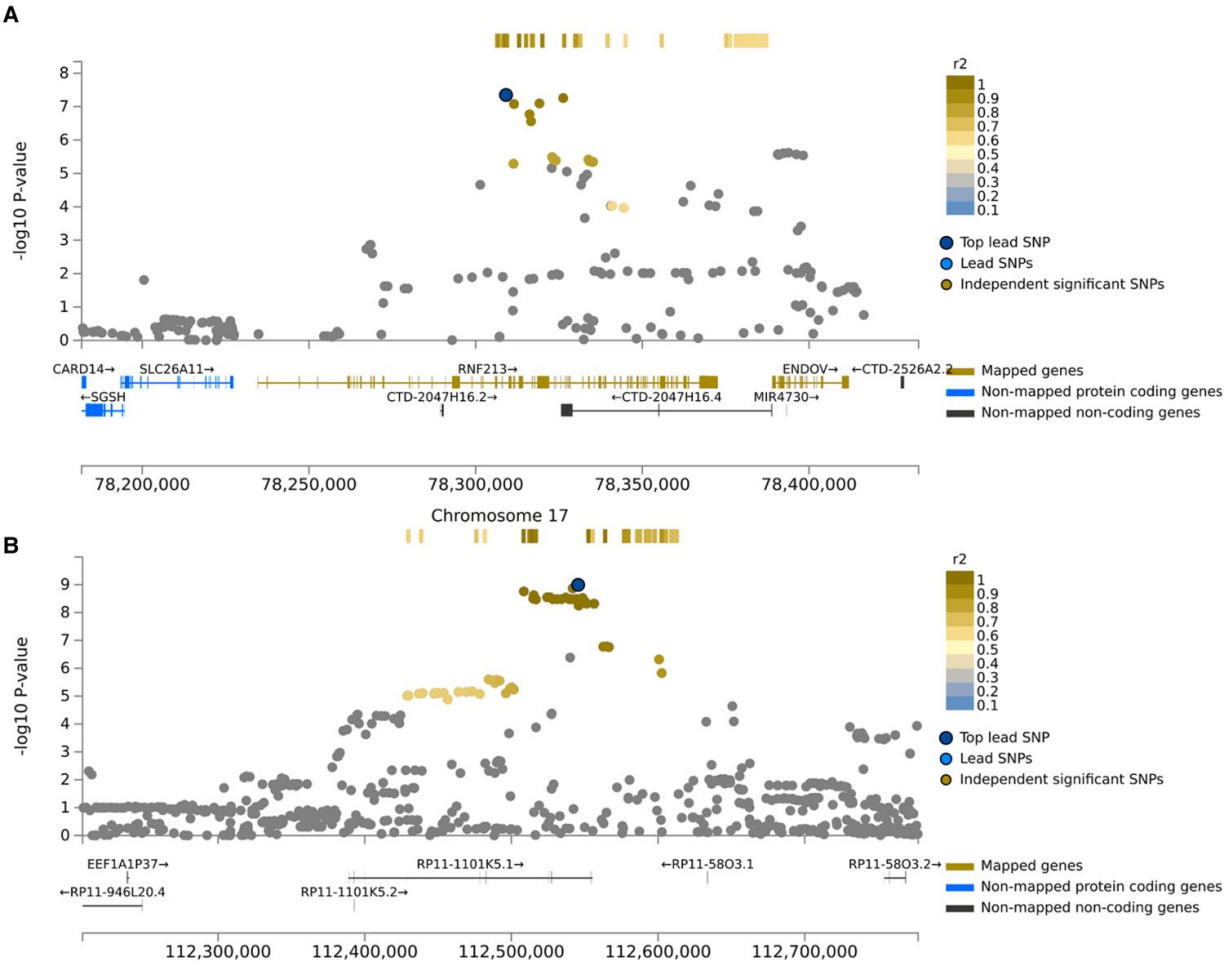
**Regional association plot.** The lead SNP (labelled by rsID) is indicated by a deep blue circle, and the *r*^2^ values of SNPs in LD with the top genotyped SNP are indicated by different colours. SNPs that are not in LD with any of the independent significant SNPs (with *r*^2^ ≤ 0.4) are grey. (**A**) Regional plot of the association of rs8072917. The −log_10_*P*-value (*y*-axis) of SNPs is presented according to their chromosomal position, Chromosome 17 position (*x*-axis). (**B**) Regional plot of the association of rs13272202. The −log_10_*P*-value (*y*-axis) of SNPs is presented according to their chromosomal position, Chromosome 8 position (*x*-axis).

Regional association plot and LD on Chromosomes 17 and 8 are presented in Fig. 4. For broadly defined headache phenotype, an SNP cluster was identified in Chromosome 17 RNF213 gene region, of which five variants rs8078851, rs9674961, rs4890009, rs8080730 and rs4890010 are in high LD (*R*^2^ > 0.93) with the top SNP rs8072917 (with lowest *P-*value of 4.49 × 10^−8^) (Fig. 4A and Supplementary Table 2). For severe headache phenotype, an SNP cluster was identified in RP11-1101K5.1 gene region. rs13272202 had the lowest *P*-value of 1.02 × 10^−9^ (Fig. 4B and Supplementary Table 2).

**Figure 5:**
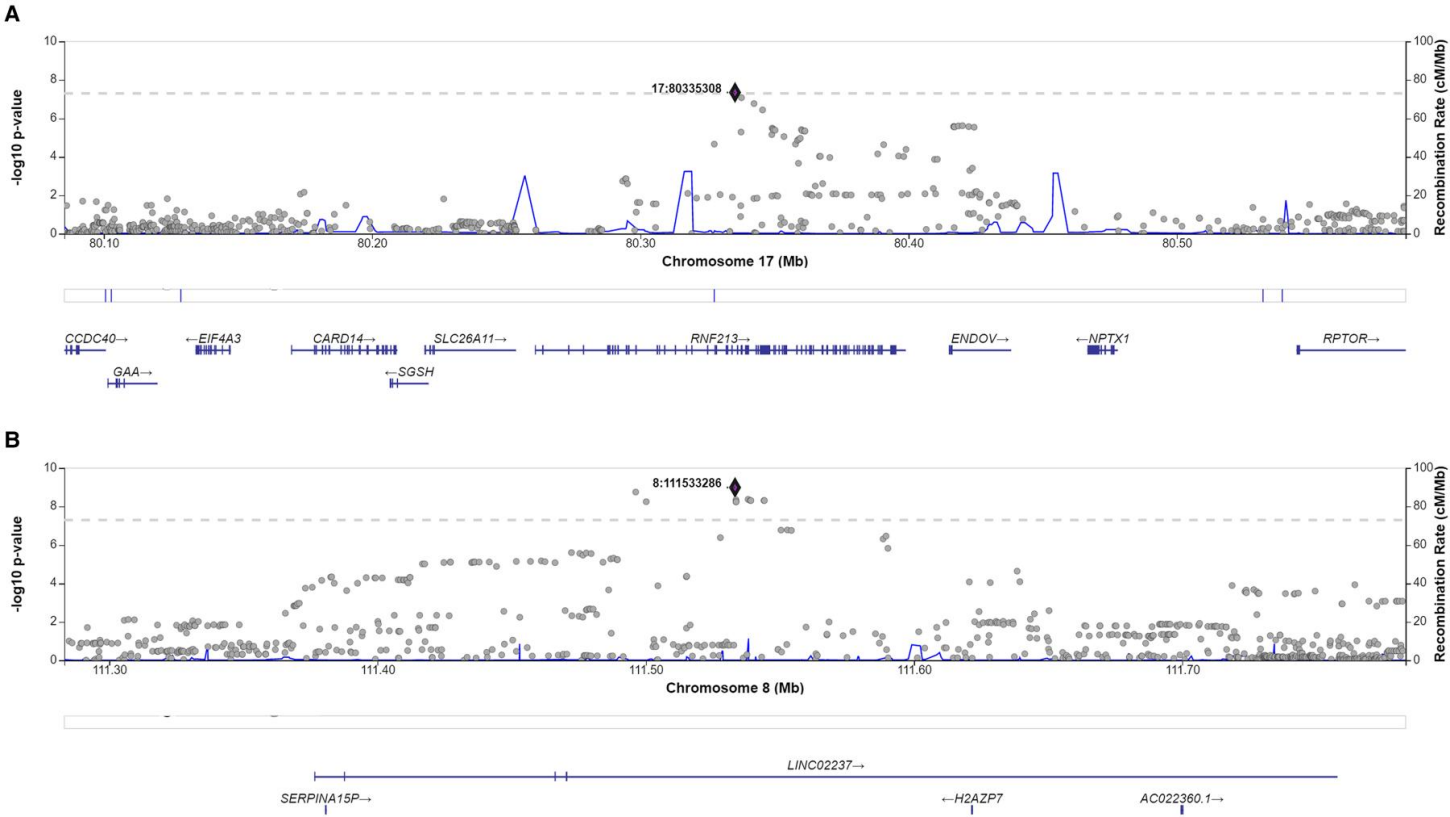
**Conditional analysis on broadly defined headaches (RNF213) (A) and severe headaches (LINC02237) (B) using the TWB cohort.** The analysis was performed using logistic regression, adjusting for age, sex and the first five population principal components. The *P*-values for the conditional association tests are 4.49 × 10^−8^ for rs80335308 on Chromosome 17 (**A**) and 1.02 × 10^−9^ for rs111533286 on Chromosome 8 (**B**). The test statistics for the conditional association tests are not reported as they are not informative for logistic regression models.

Conditional analyses were next applied to identify secondary association signals. For broadly defined headache, the conditional analysis returned that no SNPs in high LD reach genome-wide significance of *P* < 5 × 10^−8^ within the risk *loci RNF213* after conditioning on the lead variant rs8072917. This strongly indicates the accuracy of localization of the true causal variant on RNF213 gene region. For severe headache, nevertheless, eight SNPs (rs10955583, rs10087862, rs6469358, rs1115957, rs4876697, rs4876699, rs6469359 and rs7000860) still achieved genome-wide significance after conditional analysis. Though no gene region was newly identified, presence of multiple signals suggests that these associations are possibly secondary causal variants. Figure 5A and B details the regional association plots after the conditional analysis for broadly defined headaches and severe headaches.

### Fine-mapping and credible sets

**Figure 6:**
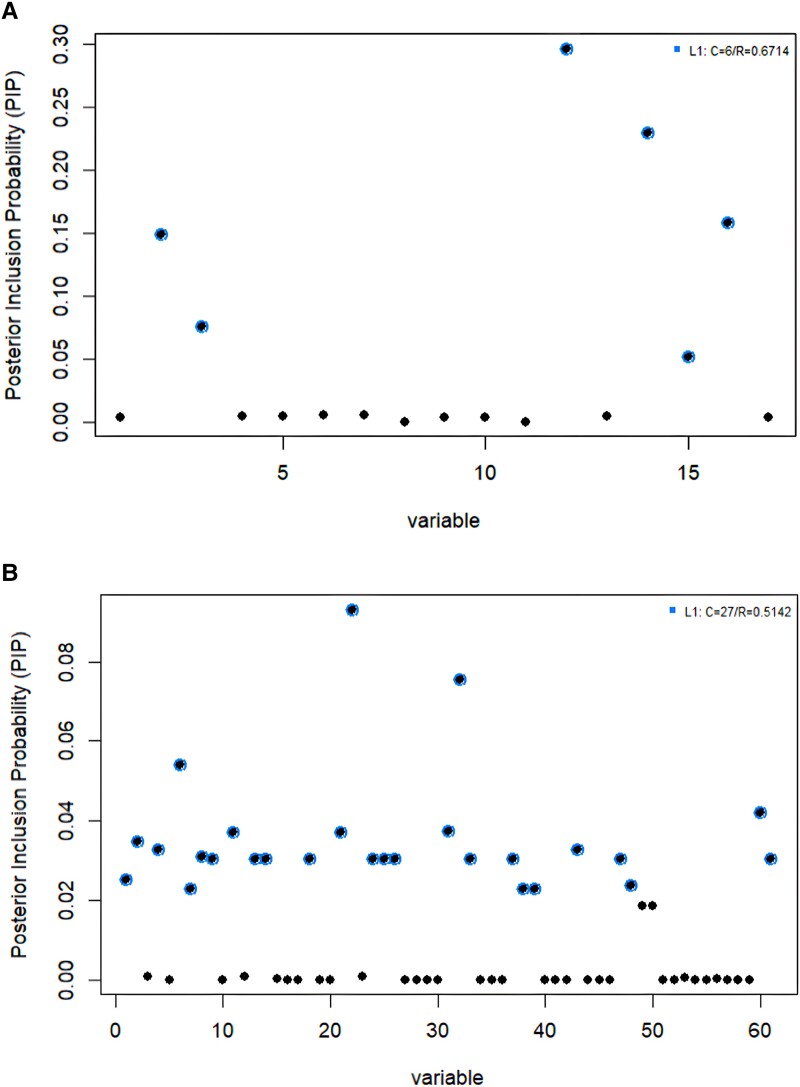
**Fine-mapping results showing the plot of PIP for broadly defined headache (A) and severe headache (B) using the TWB cohort.** The *x*-axis shows −log10 transformed *P*-values of the most associated variant, and the *y*-axis shows the PIP. The blue circles represent credible sets. Test statistic values and *P*-values are not reported in this analysis, as they are not informative for Bayesian models used in statistical fine-mapping. Statistical fine-mapping and 95% credible sets based on PIPs can be found in Table 2.

To further prioritize putative causal variants, we conducted a statistical fine-mapping analysis using susieR package. For each credible set, there was a 95% cumulative PIP that it contained at least one causal variant. Table 2 shows the 95% credible sets of potential causal variants by ranking the SNPs in descending PIP. For broadly defined headache, we identified a single credible set of genetic variants including the lead variant rs8072917 and 5 SNPs in the *loci*, with an R of 0.6714 (Fig. 6A). The index variant itself shows the highest PIP (0.297), suggesting that this is the most likely causal variant in the locus. For severe headache, 27 SNPs were identified in the 95% credible set with an R of 0.5142 (Fig. 6B). All variants in the credible set showed low PIPs, which is compatible with the interpretation of possible secondary causal variants in the conditional analysis. As an indicator of purity, R was defined as the smallest absolute correlation among the variables within the credible set.

**Table 2:** Statistical fine-mapping and 95% credible sets based on PIPs

Rsid	Chromosome	position	Beta	Standard error	PIP
Broadly defined headache					
rs8072917	17	80 335 308	0.079734968	0.01453	0.296580702
rs8078851	17	80 352 467	0.07881118	0.01449	0.229519336
rs9674961	17	80 345 336	0.077886539	0.01451	0.158301091
rs4890009	17	80 337 708	0.077886539	0.01454	0.149481231
rs4890010	17	80 342 379	0.075107472	0.01438	0.07554012
rs8080730	17	80 342 809	0.074179398	0.01441	0.051823973
Severe headache					
rs13272202	8	111 533 286	0.04287	0.261595	0.093022364
rs2195515	8	111 529 562	0.04261	0.258511	0.07556999
rs10955583	8	111 496 269	0.04263	0.256191	0.054008917
rs9297447	8	111 503 031	0.04255	0.253867	0.041972206
rs1896861	8	111 524 889	0.04256	0.253091	0.0374629
rs11776383	8	111 512 167	0.04257	0.253091	0.03716176
rs13260231	8	111 514 179	0.04257	0.253091	0.03716176
rs10093394	8	111 536 363	0.04278	0.253867	0.034867178
rs10108913	8	111 502 698	0.04259	0.252314	0.03291785
rs5894044	8	111 503 817	0.04259	0.252314	0.03291785
rs11365170	8	111 504 518	0.04293	0.253867	0.0309445
rs1160551	8	111 527 227	0.04256	0.251537	0.030356646
rs12676588	8	111 530 648	0.04256	0.251537	0.030356646
rs12678206	8	111 516 038	0.04256	0.251537	0.030356646
rs12682170	8	111 519 162	0.04256	0.251537	0.030356646
rs1429439	8	111 528 866	0.04256	0.251537	0.030356646
rs1429440	8	111 528 471	0.04256	0.251537	0.030356646
rs1579468	8	111 521 949	0.04256	0.251537	0.030356646
rs2195516	8	111 529 411	0.04256	0.251537	0.030356646
rs4876681	8	111 516 178	0.04256	0.251537	0.030356646
rs6469357	8	111 532 823	0.04256	0.251537	0.030356646
rs959495	8	111 531 363	0.04256	0.251537	0.030356646
rs10087862	8	111 538 154	0.04266	0.250759	0.025260305
rs6469358	8	111 533 630	0.04261	0.24998	0.023673069
rs1115957	8	111 539 197	0.04266	0.24998	0.022763612
rs4876697	8	111 544 080	0.04266	0.24998	0.022763612
rs4876699	8	111 544 242	0.04266	0.24998	0.022763612

### Functional annotation

SNP2GENE process in the FUMA platform was used for functional annotation and is shown in Supplementary Table 2. For broadly defined headache phenotype, we found that SNPs located within Chromosome 17 were all mapped to protein-coding gene ring finger protein 213 (*RNF213*). The majority of SNPs belonged to *RNF213* introns, and two SNPs fell within the exonic region (rs9674961 and rs4890009). For severe headache phenotype, however, no SNPs located within Chromosome 8 were mapped to protein-coding genes. These SNPs were annotated to RP11-1101K5.1 gene, an RNA gene affiliated with the long non-coding RNA (lncRNA) class.

### Gene-set analysis

Top 10 results of a MAGMA gene-set analysis are listed in Supplementary Table 3, in which several significant associations of specific biological pathways or cellular functions were revealed. Gene sets of protein complex containing pICln (CLNS1A) and Sm proteins, downregulation of MGMT gene by carmustine and regulation of cytoplasmic mitotic chromosomal structure reached a *P* < 10^−4^ but not statistical significance of *P* < 5 × 10^−6^ (0.05/10 894). More details of the gene sets can be seen in Supplementary Table 3.

### Tissue expression analysis

**Figure 7:**
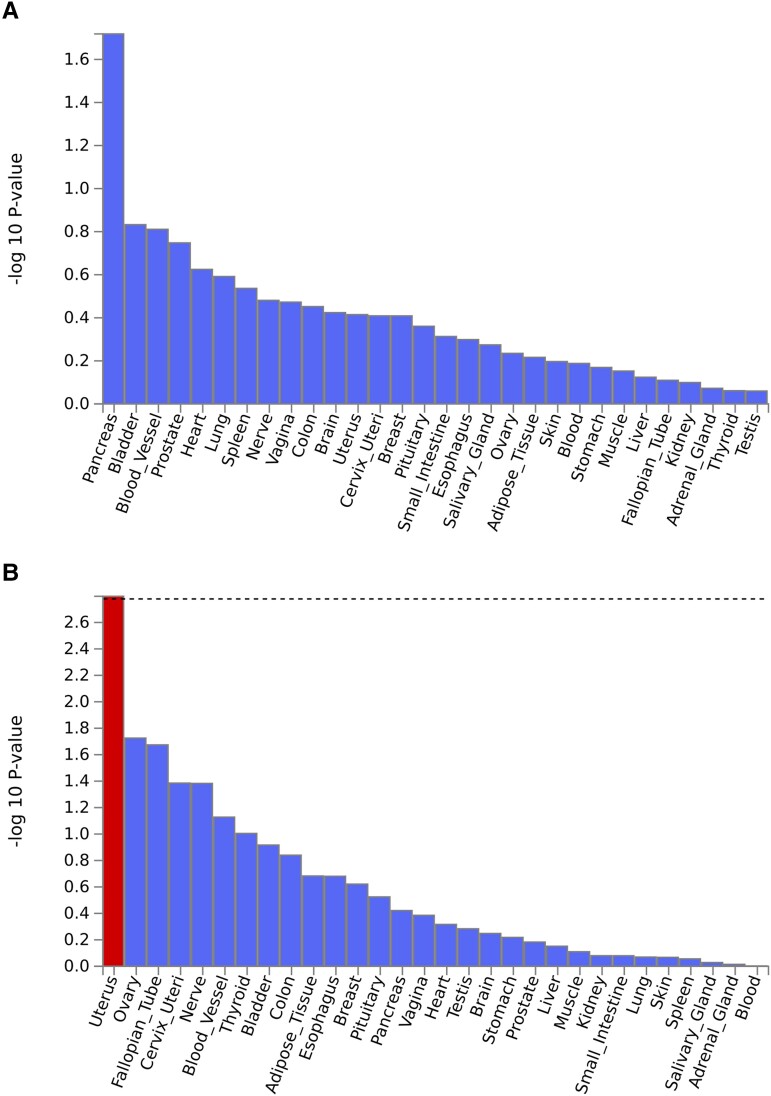
**The tissue expression results on 30 general tissue types for broadly defined headache (A) and for severe headache (B) by GTEx integrated in FUMA.** A red line indicates significance when the *P*-value of a tissue type is below 0.0017 for 30 general tissue types after Bonferroni correction for multiple hypothesis testing.

**Figure 8:**
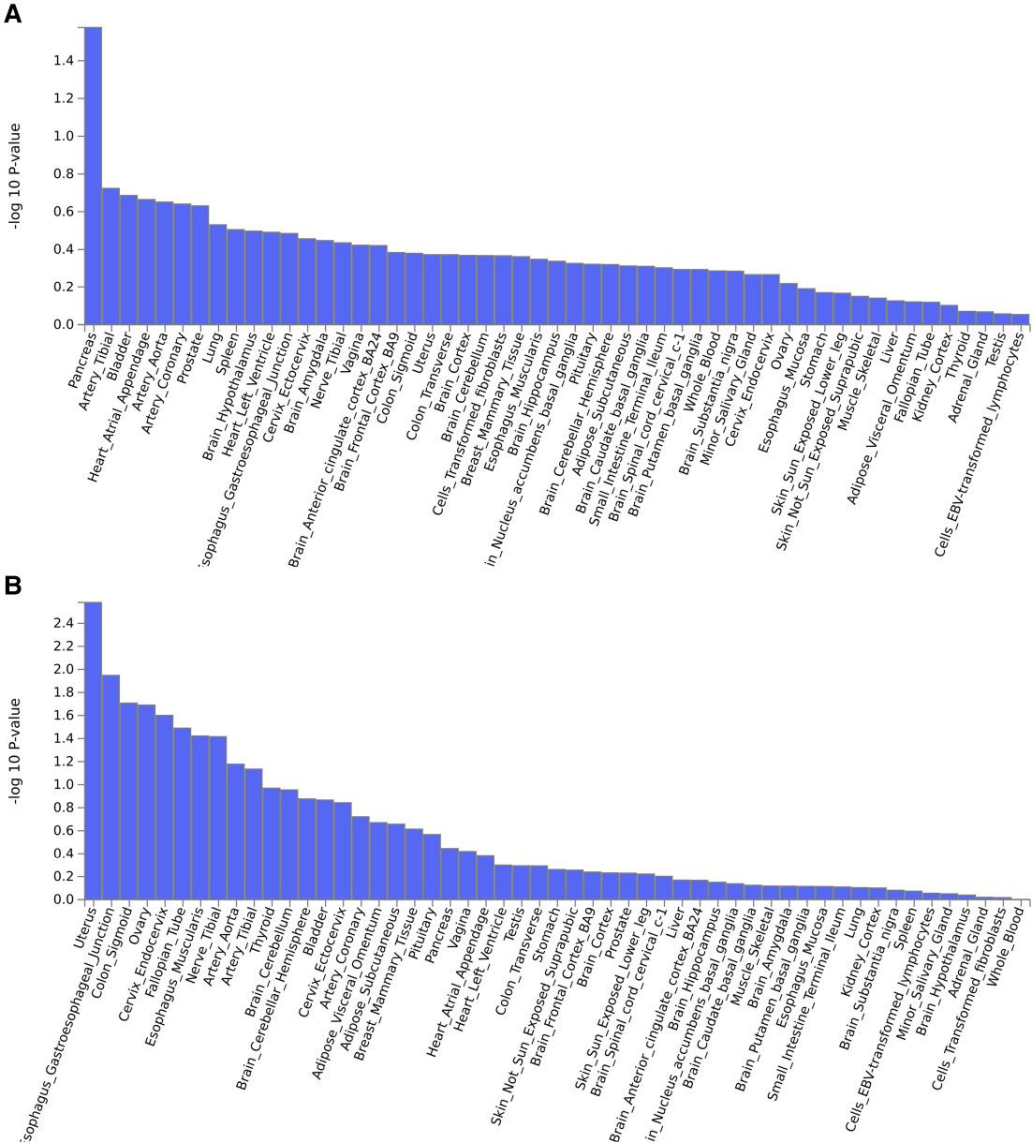
**The tissue expression results on 53 specific tissue types for broadly defined headache (A) and for severe headache (B) by GTEx integrated in FUMA.** If the *P*-value for a tissue type falls below 0.0009 for 53 specific tissue types after Bonferroni correction for multiple hypothesis testing, the tissue type is shown by a red line indicating that the tissue type is significant. The −log10 (base 10) of the *P*-values is shown on the *y*-axis. The tissue types are grouped on the *x*-axis. (**A**) For broadly defined headache, the tissue types, in order from left to right, indicating pancreas; tibial artery; bladder; heart, atrial appendage; artery aorta; artery coronary; prostate; lung; spleen; brain, hypothalamus; heart, left ventricle; oesophagus, gastro-oesophageal junction; cervix, ectocervix; brain, amygdala; nerve, tibial; vagina; brain, anterior cingulate cortex (BA24); brain, frontal cortex (BA9); colon, sigmoid; uterus; colon, transverse; brain, cortex; brain, cerebellum; cells, transformed fibroblasts; breast, mammary tissue; oesophagus, muscularis; brain, hippocampus; brain, nucleus accumbens basal ganglia; pituitary; brain, cerebellar hemisphere; adipose, subcutaneous; brain, caudate basal ganglia; small intestine, terminal ileum; brain, spinal cord cervical c-1; brain, putamen basal ganglia; whole blood; brain, substantia nigra; minor salivary gland; cervix, endocervix; ovary; oesophagus, mucosa; stomach; skin, sun exposed lower leg; skin, not sun exposed (suprapubic); muscle, skeletal; liver; adipose, visceral (omentum); fallopian tube; kidney, cortex; thyroid; adrenal gland; testis; cells, EBV-transformed lymphocytes. (**B**) For severe headache, the tissue types, from left to right, indicating uterus; oesophagus, gastro-oesophageal junction; colon, sigmoid; ovary; cervix, endocervix; fallopian tube; oesophagus, muscularis; nerve, tibial; artery, aorta; thyroid, brain, cerebellum; brain, cerebellar hemisphere; bladder; cervix, ectocervix; artery, coronary; adipose, visceral (omentum); adipose, subcutaneous; breast, mammary tissue; pituitary; pancreas; vagina; heart, atrial appendage; heart, left ventricle; testis; colon, transverse; stomach; skin, not sun exposed (suprapubic); brain, frontal cortex (BA9); brain, cortex; prostate; skin, sun exposed (lower leg); brain, spinal cord (cervical c-1); liver; brain, anterior cingulate cortex (BA24); brain, hippocampus; brain, nucleus accumbens basal ganglia; brain, caudate basal ganglia; muscle, skeletal; brain, amygdala; brain, putamen basal ganglia; oesophagus, mucosa; small intestine, terminal ileum; lung; kidney, cortex; brain, substantia nigra; spleen; cells, EBV-transformed lymphocytes; minor salivary gland; brain, hypothalamus; adrenal gland; cells, transformed fibroblasts; whole blood.

Two types of tissue analysis were performed (Figs. 7 and 8 and Supplementary Table 4). For broadly defined headache phenotype, pancreas tissue showed the lowest *P*-value in the expression analysis of 30 general tissue types (Fig. 7A), followed by several brain and vascular tissue enriched in 53 specific tissue types (Fig. 8A). For severe headache phenotype, the strongest gene enrichment was observed in uterus and other female-specific tissues in the expression analysis of 30 general tissue types (Fig. 7B), followed by gastrointestinal tract tissue, nerve tissue and vascular tissue in 53 specific tissue types (Fig. 8B). Both phenotypes demonstrate an enriched distribution of gene expression for neural tissue and vascular tissue in 30 general and 53 specific tissue types analysis, although most (except uterus tissue shown as significant by a red line in Fig. 7B) did not meet our pre-specified significance thresholds of *P* < 0.0017 (=0.05/30) for 30 general tissue types and *P* < 0.0009 (0.05/53) for 53 specific tissue types after Bonferroni correction for multiple hypothesis testing.

### Phenome-wide association studies

On the basis of the previous conditional analysis, we conducted PheWAS for the lead variants with reported associations in the UKB. For broadly defined headache, the lead variant rs8072917 was associated (FDR-adjusted *P*-value < 0.0024) with three phenotype categories: cellulitis and abscess of face and neck, cardiogenic shock and symptoms of the muscles after correction for multiple testing. For severe headache, we conducted PheWAS for the top variant rs13272202 and other eight SNPs (rs10955583, rs10087862, rs6469358, rs1115957, rs4876697, rs4876699, rs6469359 and rs7000860) that still achieved genome-wide significance after conditional analysis. Five different phenotype categories were identified to be associated (FDR-adjusted *P*-value as shown in Supplementary Figs. 1B–8J) these SNPs: vertiginous syndromes and other disorders of vestibular system, post-traumatic stress disorder (PTSD), inguinal hernia, other disease of respiratory system and jaundice (not of newborn). This result was shown in Supplementary Fig. 1A as a Manhattan plot for broadly defined headache and in Supplementary Figs. 1B–8J for severe headache.

## Discussions

In our study, we sought to identify novel genetic variations that pre-disposed individuals to headaches among 108 855 Han Chinese people including 12 026 headache cases. Among broadly defined headache phenotype, rs8072917 reached genome-wide statistical significance, and this SNP is in *cis*-acting regulatory elements of the *RNF213* and *ENDOV* gene. *RNF213* encodes E3 ubiquitin–protein ligase through protein-harbouring really interesting new gene (RING) finger domain.^29^*RNF213* has been shown to involve in angiogenesis by promoting vessel regression through inhibiting the non-canonical Wnt-signaling pathway in vascular development.^30^ It is also well known to be a susceptibility gene for Moyamoya Disease in the East Asian populations.^31,32^*RNF213* is also associated with various neurovascular conditions including intracranial atherosclerotic stenosis,^33^ coronary artery disease^34^ and systolic blood pressure,^35^ suggesting that this molecule may play an important role in vascular construction. It has been reported to play a role in modulating downstream therapeutic targets in glioblastoma tumour progression.^36^ The Endonuclease V, coded by the *ENDOV* gene, is a inosine-specific ribonuclease in RNA processing.^37^ Although its biological function remains unknown, haplotypes carrying p.R4810K in *RNF213* have been reported to be in LD with *ENDOV*.^35^

### Neurovascular mechanism of headache

It has been long debated whether headache has a vascular or a neuronal origin or whether it is a combination of both.^38^ With the conditional analysis and subsequent fine-mapping of the broadly defined headache-associated *loci*, we identified just a single credible set of *loci* with rs8072917 showing the greatest PIP. These findings supported that the lead variant rs8072917 was the true causal variant on *RNF213* gene region. The PheWAS indicated pleiotropy for this variant. These traits included cellulitis and abscess of face and neck, cardiogenic shock and symptoms of the muscles.

The role of immune system and neurogenic inflammation in primary headache has been previously studied, especially in the elucidation of migraine pathogenesis.^39,40^ The polymorphism of toll-like receptor 4, a leading receptor of innate immunity, was also associated with an increased risk of odontogenic phlegmon of the oral cavity floor, the first leading cause of facial and deep neck cellulitis.^41^ Polymorphism in *RNF213* gene was also been linked to abnormal antigen presentation and subsequent abnormal T-cell responses.^41^ Our findings may suggest a genetic pre-disposition in the regulation of the immune system that may generate susceptibility to both primary headache and infection of the face and neck. Further studies are required to validate this hypothesis and explore possible mechanisms behind these findings.

The association between primary headache disorders, mostly migraine and cardiovascular disease such as hypertension and ischaemic heart disease has been well established.^42–44^ Cardiogenic shock is a multifactorial clinical syndrome with extremely high mortality, progressing from cardiac insults preceded by different entities to the subsequent organ failure and death.^45^ Despite declining in the past two decades, acute coronary syndrome remains the leading cause of cardiogenic shock across different populations.^46,47^ Results indicated that there were shared genetic associations between headache and cardiogenic shock, in accordance with previous studies.^48^ As of association between headache and symptoms of muscle, clinically, headache attributed to temporomandibular disorders (TMD) seemed to mostly co-occur with myalgia of the temporalis.^49^ Several epidemiological studies indicated a significant overlap between myofascial TMD and TTH.^42,43^ Recent knowledge about the pathophysiology of TMD has suggested that muscle tenderness in pericranial myofascial tissues may contribute remarkably to headache attacks.^44^ There was also evidence suggesting a mutual genetic background for both muscle-related TMD and TTH pathogenesis.^50^ These findings support our PheWAS finding that headache is associated with muscle symptoms (though not clearly specified), and RNF213 functions may need further investigation.

We also identified 31 independent SNPs in Chromosome 8, led by rs13272202, to be associated with an increased risk of severe headaches. They were annotated to RP11-1101K5.1 gene, an RNA gene which is affiliated with the lncRNA class. This result is not surprising as 90% of the variants identified by GWAS are non-coding and cannot be easily linked to a candidate causal gene. Dysregulation of lncRNAs can drive tumourigenesis, and they are now considered to be associated with multiple cancer.^51^ An SNP that is not specifically linked to a functional gene has the potential to regulate gene expression via mechanisms such as modification of promoter and enhancer activity or disruption of binding sites for transcription factors in the nearby gene.

Using conditional analysis and fine-mapping, we identified eight SNPs across the severe headache *loci* (rs10955583, rs10087862, rs6469358, rs1115957, rs4876697, rs4876699, rs6469359 and rs7000860). Multiple causal signals contributed to these findings. According to our PheWAS results, these variants displayed some pleiotropy. These traits included vertiginous syndromes and other disorders of vestibular system, PTSD, inguinal hernia, other disease of the respiratory system and jaundice.

### Vestibular disorder and PTSD

Several epidemiological studies had reported a strong relationship and co-occurrence between headache, especially migraine, and vertigo due to vestibular dysfunction including BPPV or other disorders related to inner ear dysfunction.^52,53^ A recent nationwide population-based study in Taiwan showed that patients with migraine had an increased risk of developing benign paroxysmal positional vertigo when compared with controls.^54^ Another study that sought to examine the prevalence of dizziness or vertigo among migraineurs found a significant association between migraine pain severity and increased vertigo complaints.^55^ Additionally, a recent case-control study in a Chinese Han population reported a significant correlation between vestibular migraine onset and variant rs770963777 in the HTR6 gene.^56^ These results in previous studies, consistent with our findings, suggested that vestibular disorders were polygenetic and positively associated with headache.

PTSD is a stress-related psychiatric condition that typically occurs among persons exposed to traumatic events involving life threats, serious injury or death. Yet, only a small fraction of people go on to develop PTSD among individuals experiencing trauma.^57^ This is likely linked to genetic pre-disposition, and the epigenetic mechanisms in PTSD were previously examined.^58^ PTSD is also highly co-morbid with chronic pain conditions including migraine and tension headaches.^59^ A recent study of monozygotic twins found several epigenetic changes shared by PTSD and migraine, suggesting that genetic, environmental and epigenetic factors determined a person’s susceptibility to both conditions.^60^ Together with PTSD traits identified in our PheWAS, our findings suggested common variants that might contribute to genetic susceptibility to headache severity and PTSD through epigenetic pathway. Further research is needed to validate these hypotheses.

Gormley *et al.*’s^17^ meta-analysis of 22 migraine GWAS identified 16 SNPs in the RNF213 locus, two of which representing missense mutations. In the pathway and tissue expression enrichment analyses, their findings reported strong evidence that vascular dysfunction is important in migraine susceptibility. Indeed, some migraine-associated genes have previously been associated with vascular disease (PHACTR1, TGFBR2, LRP1, PRDM16, RNF213, JAG1, HEY2, GJA1 and ARMS2).

Interestingly, evidence from tissue expression analysis of GWAS of Meng *et al.*^18^ showed that brain function rather than vascular tissue was more related to broadly defined headache, which was different from the predominant theory of vascular aetiology of migraine. In our findings, both tissue expression analyses on 30 general tissue types and 53 specific tissue types show an enriched distribution of gene expression in neural tissue and vascular tissue. On the basis of all results together, we demonstrated that both neuronal and vascular factors are involved in the headache mechanism.

Of note, a previous study of mutation genotypes of RNF213 gene from moyamoya patients in Taiwan reported clinical features of migraine or headache in 27.8% of patients.^61^ In summary, our results replicate the findings of previous studies for gene *RNF213* and provide novel associations of gene *ENDOV* with broadly defined headache phenotype in the Han Chinese population. The replication of *RNF213* identified in GWAS from Western countries validates our broadly defined headache definition and suggests a shared genetic basis across ethnic groups. The available studies to support heritability in Asian families for migraine or headache are limited. However, there is some evidence to support the heritability in the aspects of differences in the prevalence across ethnic background and familial aggregation with early onset and anticipation. As mentioned above, the prevalence of migraine is higher in Western populations than that in Asians.^7^ Additionally, one study that investigated the common genetic variant load in 8319 participants from 1589 families in Finland showed that the polygenic load is associated with a lower age at onset and severity of migraine.^62^ Based on these results, the inheritance depending on ethnicity and regional difference may suggest a combined genetic and environmental contribution to headache. We were not able to replicate associations for the other SNPs, and possible reasons include the difference in the target populations and study outcomes examined. In our study, the headache cases were divided into subgroups according to their severity. However, findings in this study have provided a discovery phase of candidate genes to fulfil the much needed GWAS effort on populations of non-European ancestry.

### Limitations

This study has several limitations. First, TWB only included individuals aged 30–70 years and self-reported Taiwanese Han Chinese ancestry. We therefore had no data for people aged from 18 to 29 years old and may not accurately estimate the headache prevalence for the general population. However, a previous study suggested the prevalence of headache was similar to that in our study.^63^ Second, patients diagnosed with cancer have been excluded from our study samples. Although the two novel genes we found have been proven to possess oncogenic potential, we could not evaluate their association with prevalence of cancer or molecular mechanism. In addition, the questionnaire-based headache diagnosis does not reflect the contemporary knowledge on the pathophysiology of headache and, as a result, may be combining headaches of distinctive aetiologies into a single category. It is unlikely that all participants were examined by a neurologist in a population-based survey of this size. The validation of the questionnaire is not feasible due to de-identification of all participants. Retrospective self-reporting of headache severity may have led to recall bias and underestimation of disease prevalence due to misclassification. Despite the lack of detailed specificity and sensitivity derived from other validation studies, the questionnaire is still comparable with the one in the UKB study.^18^ Although not exactly the same, both our study and the UKB study define headache with similar pain-related questionnaires and ‘headache or migraine’ together as one option is offered in both questionnaires. Thus, the selection bias towards migraine or migraine-like headache is less likely. PheWAS was conducted with UKB instead of TWB because TWB lacked comprehensive phenotypes. To extend generalizability, we examined different biobanks and compared our results with those of other countries and ethnicities. However, environmental interactions complicate the search for genomic explanations for any given disease or phenotype, especially for people living in different countries with different environmental and lifestyle factors. The compatibility between two different ethnicities should still be interpreted cautiously.

Different phenotypes of headache may share common genetic susceptibility, as discovered by a previous work based on UKB. Further functional studies and more detailed phenotyping questions are needed to elucidate the genetic components of the traits we found and clarify their underlying biological pathways.

## Conclusion

Our study has identified 33 genetic *loci* for headaches using GWAS in the Han Chinese population in Taiwan. Combining results from GWAS and FUMA together, we highlighted the role of *RNF213* gene and *ENDOV* gene play in the biological mechanism of broadly defined headache. It may help contribute to the development of a target intervention and to form a foundation of future polygenic scores in headache specific among individuals of East Asian ancestry. Using genetic data linked to electronic health records from different countries, our study can be replicated thereby establishing polygenic risk scores, and our findings can therefore have an impact on a broad spectrum of ethnicities worldwide. Through our genetic study of headache, genetic tests may be developed to identify genetic risk factors that may be offset by modifiable lifestyle factors. Our GWAS and PheWAS also reveal genotype–phenotype associations with headache, which will facilitate the development of novel drug mechanism pathways and targets.

## Supplementary Material

fcad167_Supplementary_Data

## Data Availability

The data sets generated and/or analysed during the current study are not publicly available due to the data confidentiality requirements of the ethics committee but are available from the corresponding author on reasonable request and approval from the ethics committee.

## Abbreviations

FDR =: false discovery rate
GWAS =: genome-wide association studies
HIT-6 =: six-item Headache Impact Test
IBD =: identity by descent
IBS =: identity by state
MAF =: minor allele frequency
MIDAS =: Migraine Disability Assessment
MSQ =: Migraine-Specific Questionnaire
MTOQ =: Migraine Treatment Optimization Questionnaire
PheWAS =: phenome-wide association studies
PROM =: patient-reported outcome measures
Q–Q plots =: quantile–quantile plots
SNP =: single-nucleotide polymorphism
TTH =: tension-type headache
TWB =: Taiwan Biobank
YLD =: years lived with disability
