# Supplementary material for: Genome–phenome wide association study of broadly defined headache
Source: Brain Commun. 2023 May 24;5(3):fcad167. doi: 10.1093/braincomms/fcad167 (PMC10243784; doi:10.1093/braincomms/fcad167)
Supplement: fcad167_Supplementary_Data [file fcad167_supplementary_data.pdf]

Genome–Phenome Wide Association Study of Broadly-Defined Headache

*[1]Wan-Ting Hsu MS, *[2]Yu-Ting Lee MD, [2]Jasmine Tan,[3,4] Yung-Han Chang BSc,[5]Frank Qian MD, MPH, [2]Kuei-Yu Liu MD, [6]Jo-Ching Hsiung MD, [7]Chia-Hung Yo, MD, [8]Sung-Chun Tang MD, PhD, [1,9]Xia Jiang MD, PhD, *[2,3,10]Chien-Chang Lee MD, ScD
[1]Department of Epidemiology, Harvard T.H. Chan School of Public Health, Boston, MA, USA
[2]Health Data Science Research Group, National Taiwan University Hospital, Taipei, Taiwan
[3]Department of Emergency Medicine, National Taiwan University Hospital, Taipei, Taiwan
[4]Department of Statistics, University of California, Los Angeles, Los Angeles, USA
[5]Department of Medicine, Beth Israel Deaconess Medical Center, Harvard Medical School, Boston, MA, USA
[6]Department of Pediatrics, Einstein Medical Center-Philadelphia, Philadelphia, PA, USA
[7]Department of Emergency Medicine, Far Eastern Memorial Hospital, New Taipei City, Taiwan
[8]Department of Neurology, National Taiwan University Hospital, Taipei, Taiwan
[9]Department of Clinical Neuroscience, Center for Molecular Medicine, Karolinska Institutet, Stockholm, Sweden
[10]The Center for Intelligent Healthcare, National Taiwan University Hospital, Taipei, Taiwan

*Wan-Ting Hsu and Yu-Ting Lee contributed equally to this work.
Correspondence to:
Chien-Chang Lee, MD, ScD (Harvard)
Health Data Science Research Group, National Taiwan University Hospital
The Center for Intelligent Healthcare, National Taiwan University Hospital
Department of Emergency Medicine, National Taiwan University Hospital
No.7, Chung Shan S. Rd., Zhongzheng Dist., Taipei City 100, Taiwan.
Email: hit3transparency@gmail.com
TEL: +886-2-2312-3456 ext. 63485

Supplementary Figure 1. Manhattan plot of phenome-wide association studies (PheWAS) results for headache SNPs using the UK Biobank. Each plot is stratified by disease group and within each disease group, we report the results related to rs8072917. Results were adjusted for 5 PCs. Camel brown, light blue and gray lines indicate the phenome-wide, the suggestive and the nominal significance thresholds. (P = 3×10-5, p = 0.0024 and p = 0.05, respectively)**(A)**; rs13272202 for severe headache (5 PCs, 1 top SNP + 8 causal variants, p threshold= 0.05, minimum p value= 0.0005), the suggestive and the nominal significance thresholds are p = 0.00000328, p = 0.0023 and p = 0.05 respectively**(B)**, rs10087862 the suggestive and the nominal significance thresholds are p =0.00003, p = 0.00026 and p = 0.05 respectively**(C)**; rs10955583 the suggestive and the nominal significance thresholds are p=0.00003, p = 0.00022 and p = 0.05 respectively(**D)**; rs1115957 the suggestive and the nominal significance thresholds are p = 0.00003, p = 0.00023 and p = 0.05 respectively**(E)**; rs4876697 the suggestive and the nominal significance thresholds are p = 0.00003, p = 0.00022 and p = 0.05 respectively**(F)**; rs4876699 the suggestive and the nominal significance thresholds are p = 0.00003, p = 0.00022 and p = 0.05 respectively**(G);** rs6469358 the suggestive and the nominal significance thresholds are p = 0.00003, p = 0.00022 and p = 0.05 respectively**(H);** rs6469358 the suggestive and the nominal significance thresholds are p = 0.00003, p = 0.00022 and p = 0.05 respectively**(I);** and rs6469359 the suggestive and the nominal significance thresholds are p = 0.00003, p = 0.00022 and p = 0.05 respectively**(J)**.

Supplementary Figure 1A. Headache (rs8072917) PheWAS Manhattan plot (5 PCs, p threshold= 0.05, minimum p-value= 0.0003). The PheWAS Manhattan plot displays the association of the rs8072917 variant with various phenotypes, with a p-value threshold of 0.05 and a minimum p-value of 0.0003, using 5 principal components (PCs). The -log10 (base 10) of the p-values is shown on the y-axis. The phenotype groups are color-coded and grouped on the x-axis, from left to right, indicating infectious diseases, neoplasms, endocrine/metabolic disorders, hematopoietic diseases, mental disorders, neurological disorders, disorders of sense organs, circulatory system diseases, respiratory diseases, digestive diseases, genitourinary diseases, pregnancy complications, dermatologic diseases, musculoskeletal diseases, congenital anomalies, symptoms, and injuries & poisoning.

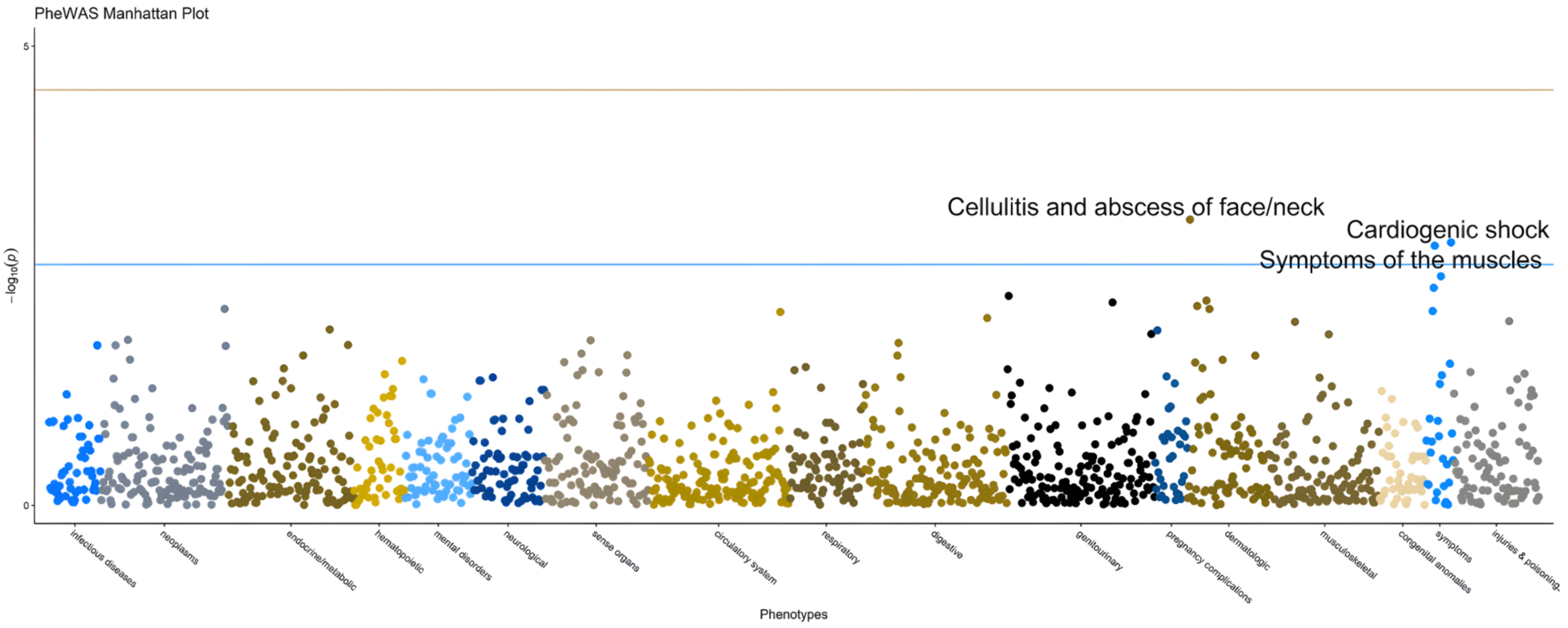

Supplementary Figure 1B. PheWAS Manhattan plot for rs13272202, with 1 top SNP and 8 causal variants, for severe headache. The analysis was performed with a p-value threshold of 0.05 and a minimum p-value of 0.0005, using 5 principal components (PCs). The Bonferroni-corrected significance threshold is 0.00000328, and the false discovery rate (FDR) is 0.0023, with a nominal p-value of 0.05. The -log10 (base 10) of the p-values is shown on the y-axis. The phenotype groups are color-coded and grouped on the x-axis, from left to right, indicating infectious diseases, neoplasms, endocrine/metabolic disorders, hematopoietic diseases, mental disorders, neurological disorders, disorders of sense organs, circulatory system diseases, respiratory diseases, digestive diseases, genitourinary diseases, pregnancy complications, dermatologic diseases, musculoskeletal diseases, congenital anomalies, symptoms, and injuries & poisoning.

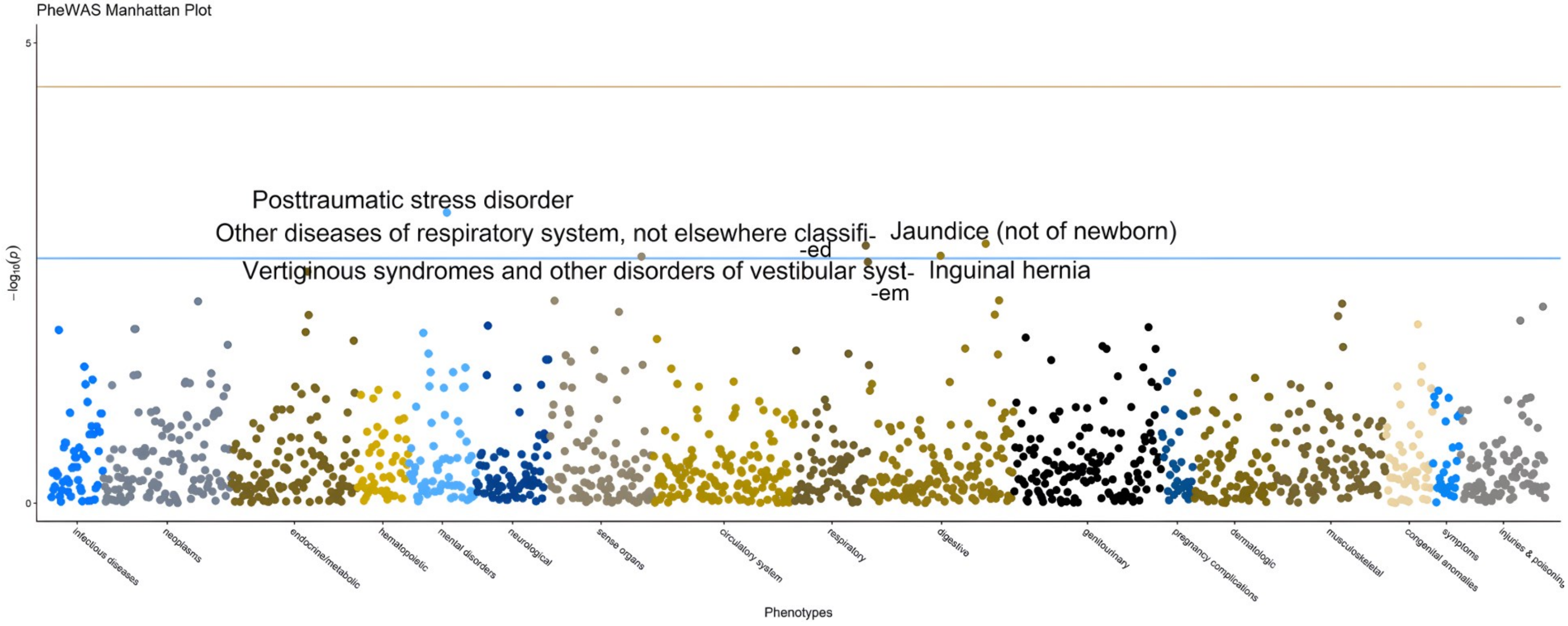

Supplementary Figure 1C. PheWAS Manhattan plot for rs10087862. Bonfferoni:0.00003. FDR: 0.00026. P-value: 0.05. The -log10 (base 10) of the p-values is shown on the y-axis. The phenotype groups are color-coded and grouped on the x-axis, from left to right, indicating infectious diseases, neoplasms, endocrine/metabolic disorders, hematopoietic diseases, mental disorders, neurological disorders, disorders of sense organs, circulatory system diseases, respiratory diseases, digestive diseases, genitourinary diseases, pregnancy complications, dermatologic diseases, musculoskeletal diseases, congenital anomalies, symptoms, and injuries & poisoning.

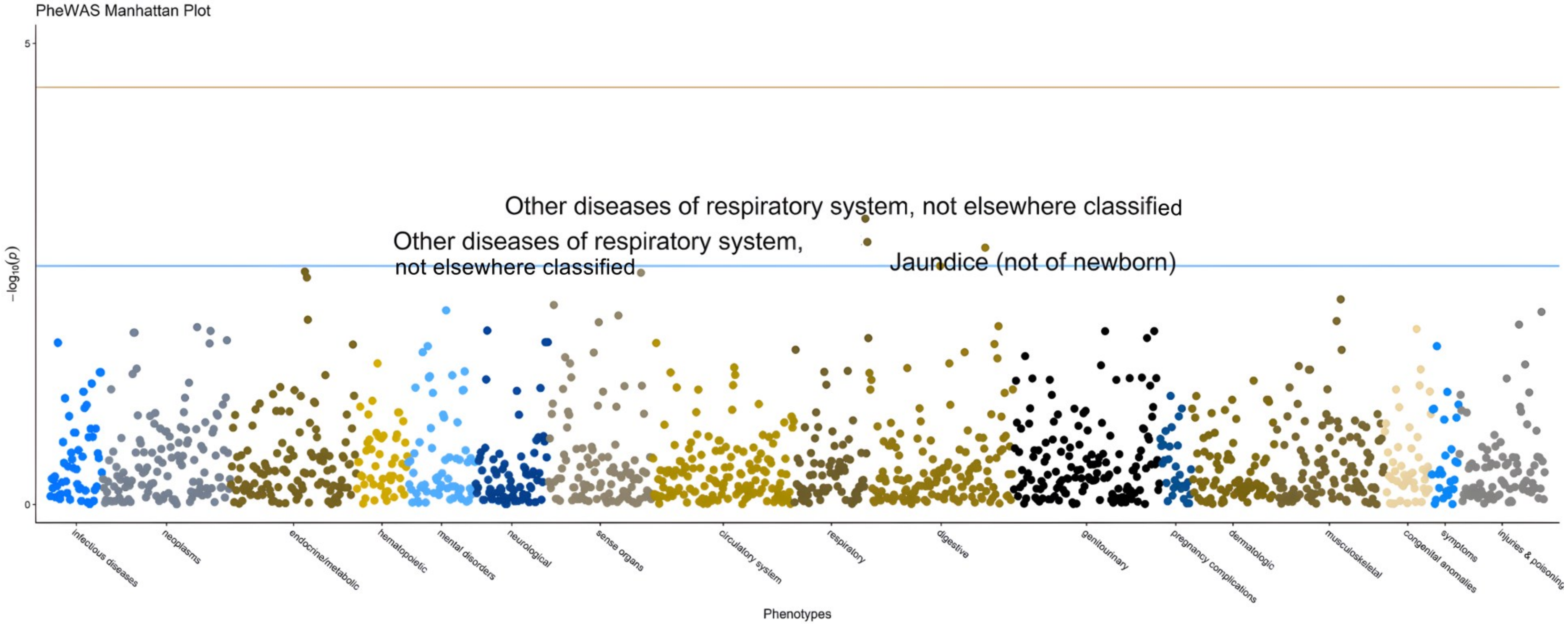

Supplementary Figure 1D. PheWAS Manhattan plot for rs10955583. Bonfferoni:0.00003. FDR: 0.00022. *P*-value: 0.05. The -log10 (base 10) of the p-values is shown on the y-axis. The phenotype groups are color-coded and grouped on the x-axis, from left to right, indicating infectious diseases, neoplasms, endocrine/metabolic disorders, hematopoietic diseases, mental disorders, neurological disorders, disorders of sense organs, circulatory system diseases, respiratory diseases, digestive diseases, genitourinary diseases, pregnancy complications, dermatologic diseases, musculoskeletal diseases, congenital anomalies, symptoms, and injuries & poisoning.

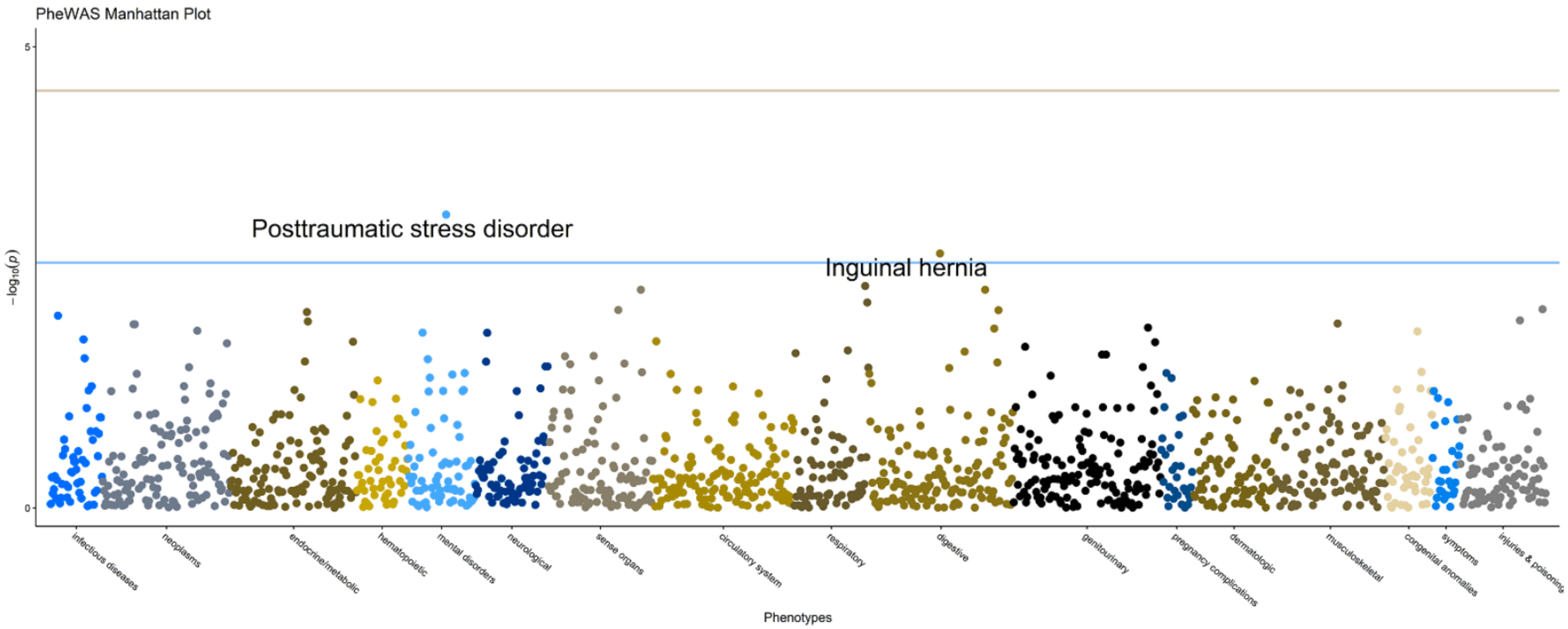

Supplementary Figure 1E. PheWAS Manhattan plot for rs1115957. Bonferroni:0.00003. FDR: 0.00023. *P*-value: 0.05. The -log10 (base 10) of the p-values is shown on the y-axis. The phenotype groups are color-coded and grouped on the x-axis, from left to right, indicating infectious diseases, neoplasms, endocrine/metabolic disorders, hematopoietic diseases, mental disorders, neurological disorders, disorders of sense organs, circulatory system diseases, respiratory diseases, digestive diseases, genitourinary diseases, pregnancy complications, dermatologic diseases, musculoskeletal diseases, congenital anomalies, symptoms, and injuries & poisoning.

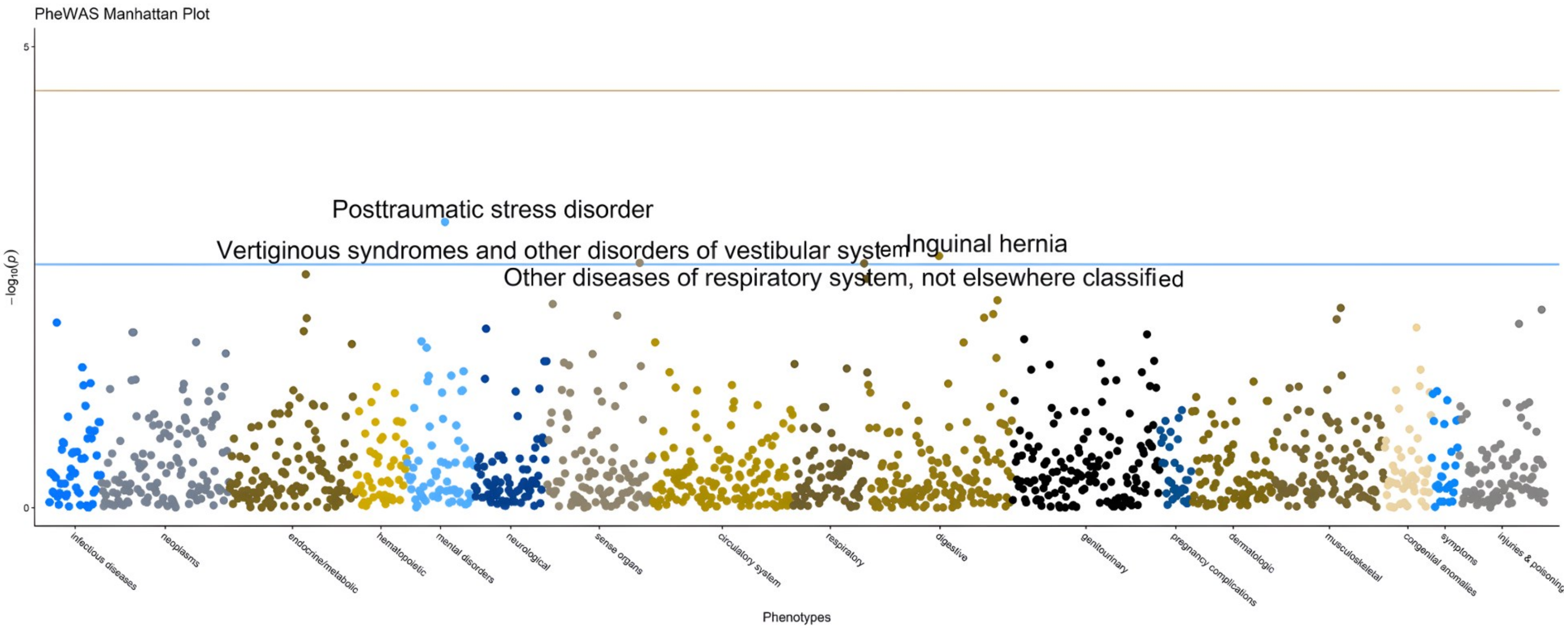

Supplementary Figure 1F. PheWAS Manhattan plot for rs4876697. Bonferroni:0.00003. FDR: 0.00022. *P*-value: 0.05. The -log10 (base 10) of the p-values is shown on the y-axis. The phenotype groups are color-coded and grouped on the x-axis, from left to right, indicating infectious diseases, neoplasms, endocrine/metabolic disorders, hematopoietic diseases, mental disorders, neurological disorders, disorders of sense organs, circulatory system diseases, respiratory diseases, digestive diseases, genitourinary diseases, pregnancy complications, dermatologic diseases, musculoskeletal diseases, congenital anomalies, symptoms, and injuries & poisoning.

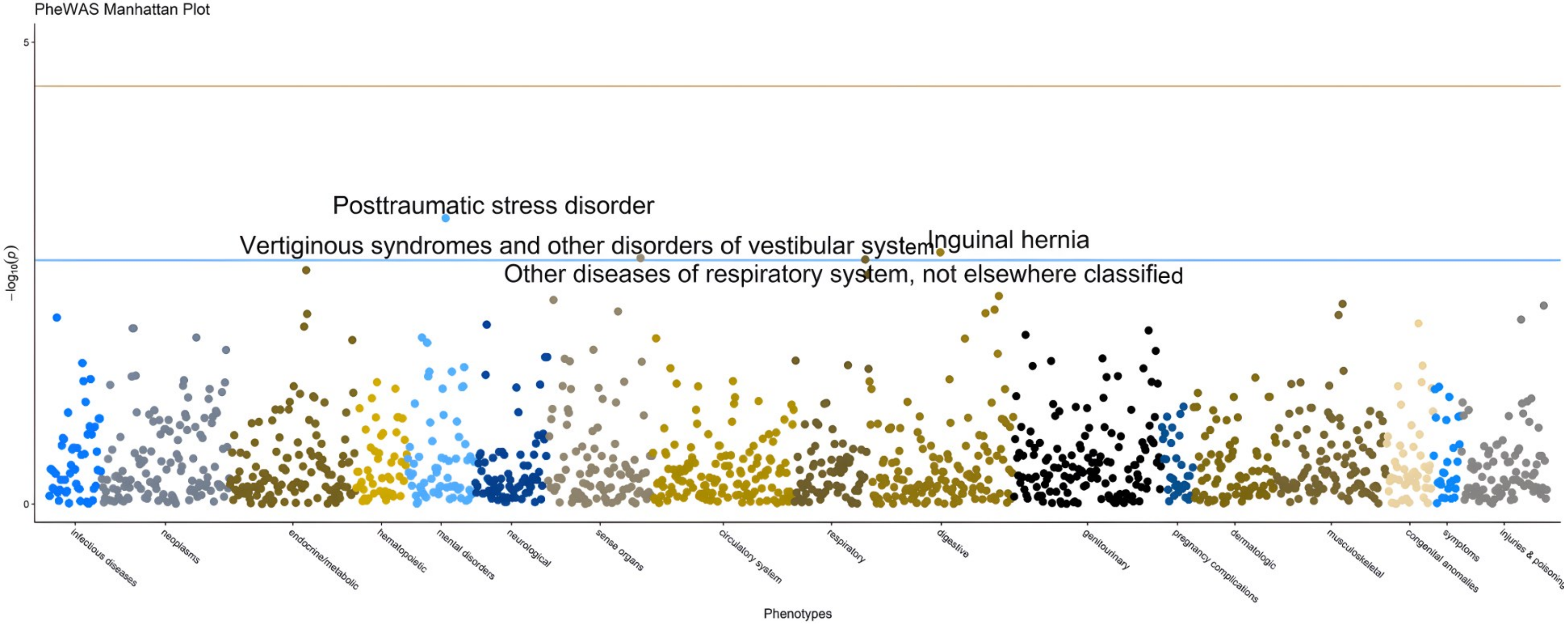

Supplementary Figure 1G. PheWAS Manhattan plot for rs4876699. Bonferroni:0.00003. FDR: 0.00022. *P*-value: 0.05. The -log10 (base 10) of the p-values is shown on the y-axis. The phenotype groups are color-coded and grouped on the x-axis, from left to right, indicating infectious diseases, neoplasms, endocrine/metabolic disorders, hematopoietic diseases, mental disorders, neurological disorders, disorders of sense organs, circulatory system diseases, respiratory diseases, digestive diseases, genitourinary diseases, pregnancy complications, dermatologic diseases, musculoskeletal diseases, congenital anomalies, symptoms, and injuries & poisoning.

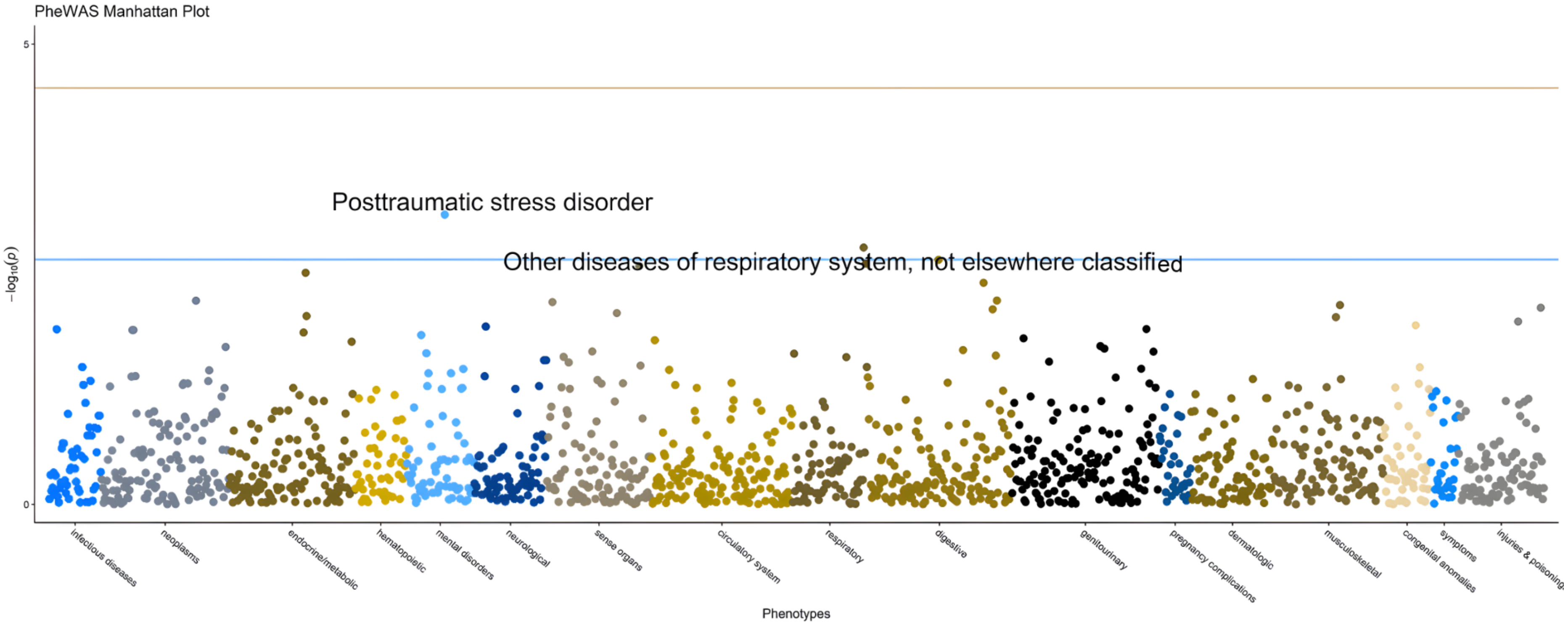

Supplementary Figure 1H. PheWAS Manhattan plot for rs6469358. Bonferroni:0.00003. FDR: 0.00022. *P*-value: 0.05. The -log10 (base 10) of the p-values is shown on the y-axis. The phenotype groups are color-coded and grouped on the x-axis, from left to right, indicating infectious diseases, neoplasms, endocrine/metabolic disorders, hematopoietic diseases, mental disorders, neurological disorders, disorders of sense organs, circulatory system diseases, respiratory diseases, digestive diseases, genitourinary diseases, pregnancy complications, dermatologic diseases, musculoskeletal diseases, congenital anomalies, symptoms, and injuries & poisoning.

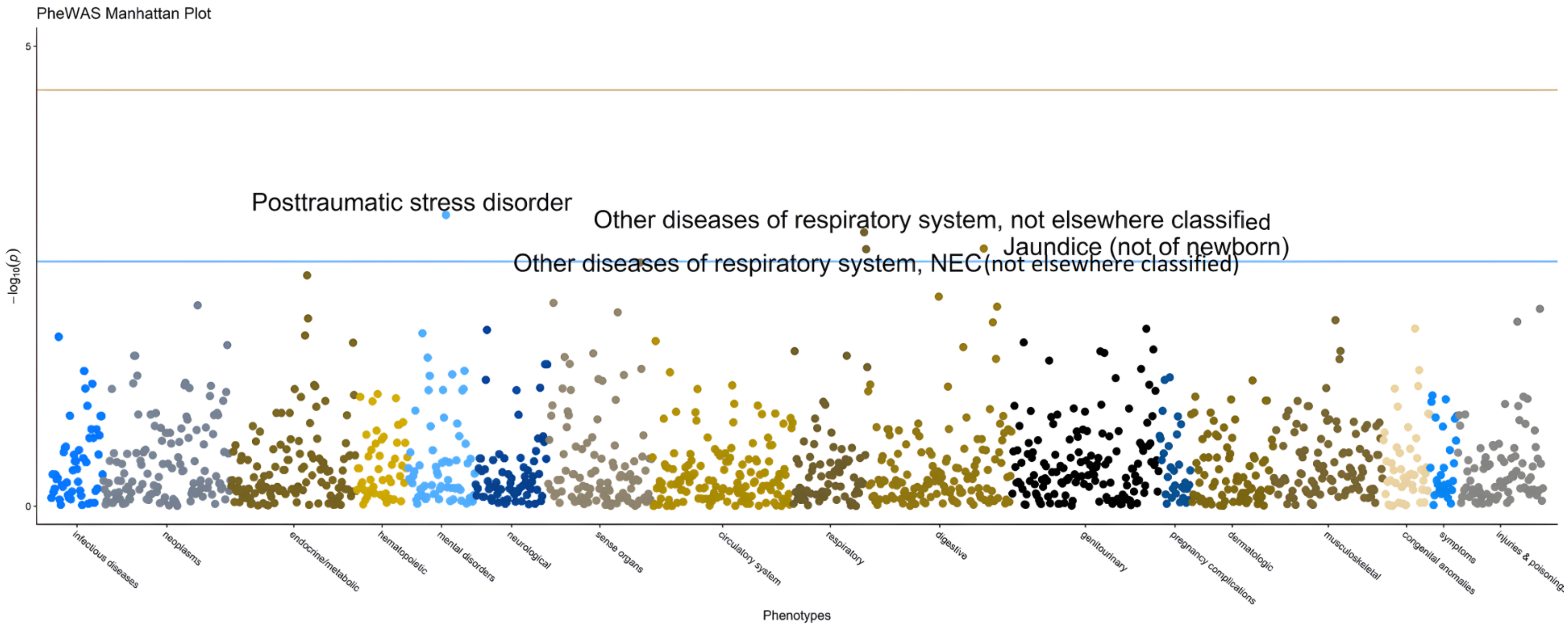

Supplementary Figure 1I. PheWAS Manhattan plot for rs6469359. Bonfferoni:0.00003. FDR: 0.00022. *P*-value: 0.05. The -log10 (base 10) of the p-values is shown on the y-axis. The phenotype groups are color-coded and grouped on the x-axis, from left to right, indicating infectious diseases, neoplasms, endocrine/metabolic disorders, hematopoietic diseases, mental disorders, neurological disorders, disorders of sense organs, circulatory system diseases, respiratory diseases, digestive diseases, genitourinary diseases, pregnancy complications, dermatologic diseases, musculoskeletal diseases, congenital anomalies, symptoms, and injuries & poisoning.

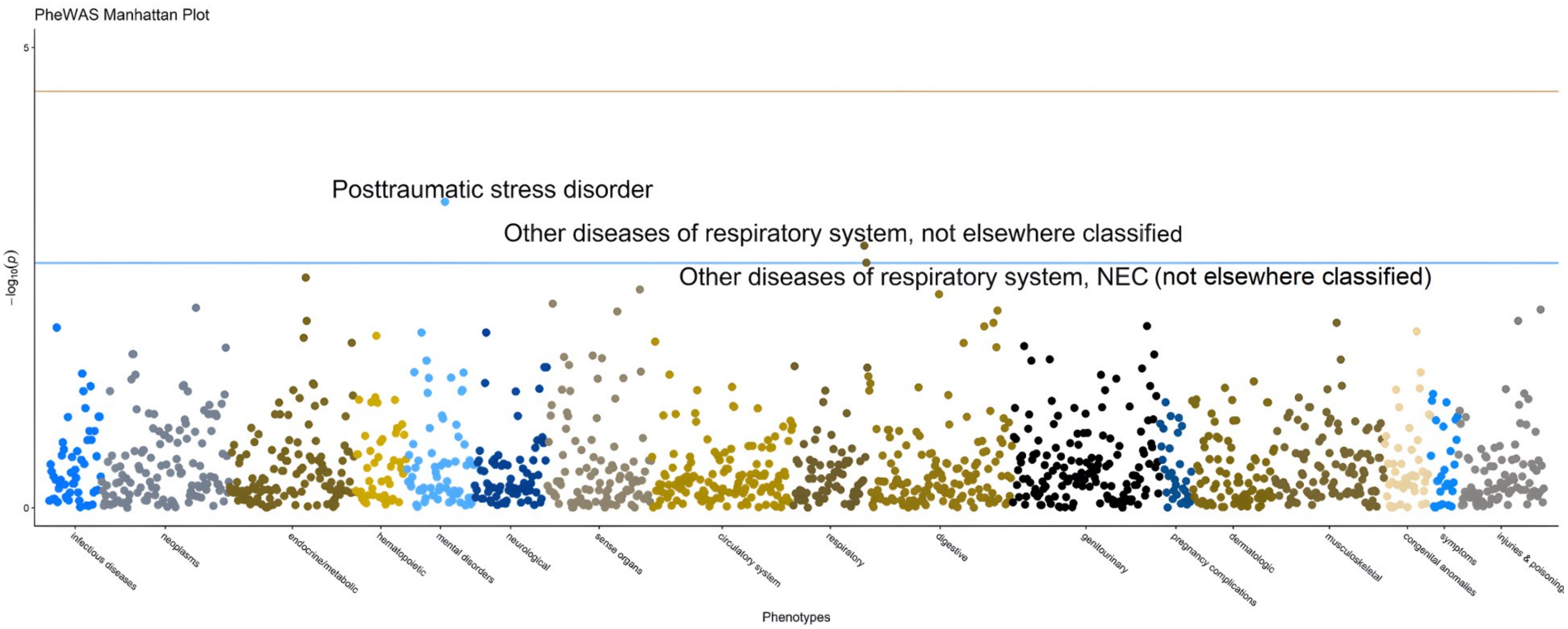

Supplementary Figure 1J. PheWAS Manhattan plot for rs7000860. Bonfferoni:0.00003. FDR: 0.00022. *P*-value: 0.05. The -log10 (base 10) of the p-values is shown on the y-axis. The phenotype groups are color-coded and grouped on the x-axis, from left to right, indicating infectious diseases, neoplasms, endocrine/metabolic disorders, hematopoietic diseases, mental disorders, neurological disorders, disorders of sense organs, circulatory system diseases, respiratory diseases, digestive diseases, genitourinary diseases, pregnancy complications, dermatologic diseases, musculoskeletal diseases, congenital anomalies, symptoms, and injuries & poisoning.

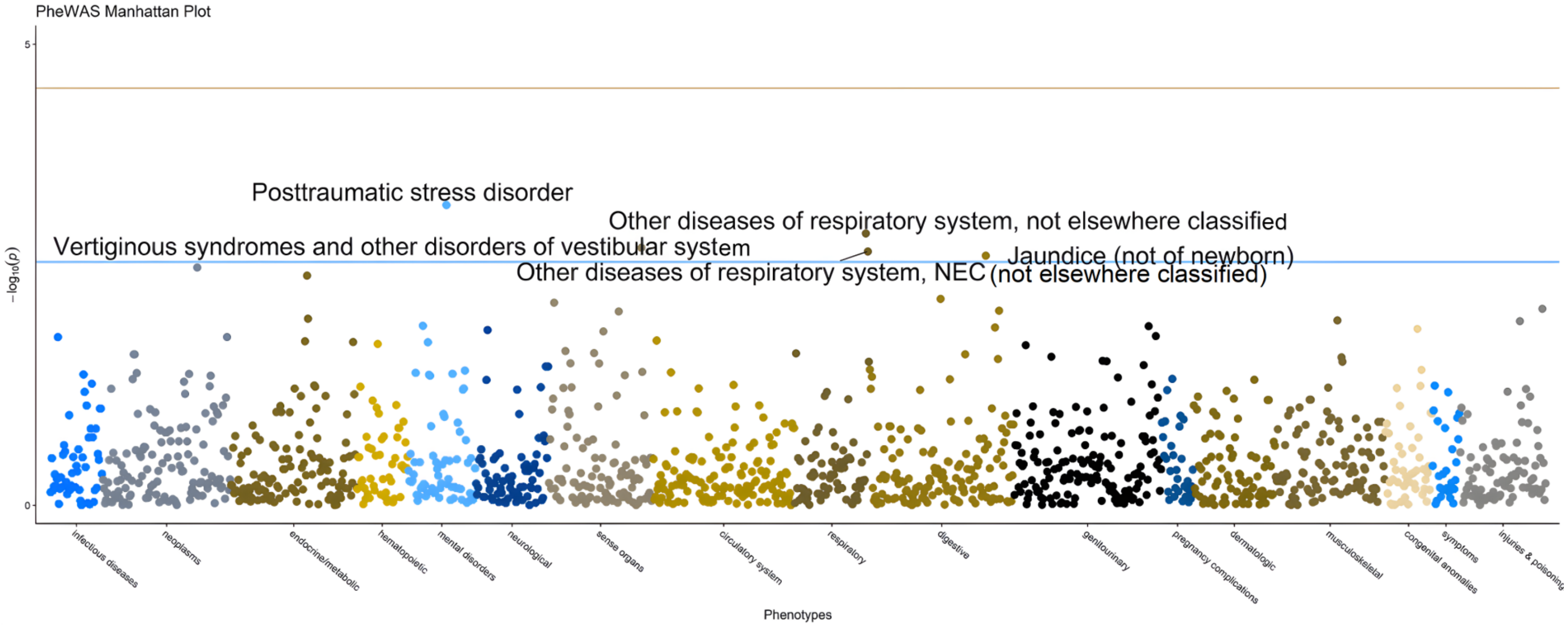

Supplementary Table 1. Results of association analyses of headache

			Cases				*Controls*				*Additive*[b]		*Dominant*[b]		Recessive		Conditional Analysis
SNP	Chr	Allele 1/2[a]	11	12	22	RAF	11	12	22	*RAF*	*P*-value	Risk allele OR (95% CI)	*P*-value	Risk allele OR (95% CI)	*P*-value	Risk allele OR (95% CI)	P-value or LD
**Headache**																	
rs8072917	17	T	1638	5601	4599	0.3749	12119	43703	39321	0.357	4.49E-08	1.083(1.052-1.114)	2.03E-07	1.111(1.068-1.157)	0.00058	1.104(1.044-1.169)	Top SNP
rs8078851	17	T	1632	5632	4651	0.3733	12119	43886	39707	0.3559	5.54E-08	1.082(1.052-1.113)	2.12E-07	1.111(1.068-1.156)	0.000744	1.102(1.042-1.167)	0.1487
rs9674961	17	A	1634	5621	4630	0.374	12149	43873	39534	0.3567	8.06E-08	1.081(1.051-1.112)	3.16E-07	1.109(1.066-1.154)	0.000811	1.102(1.041-1.166)	0.349
rs4890009	17	A	1624	5592	4621	0.3734	12042	43603	39443	0.3559	8.38E-08	1.081(1.051-1.112)	3.19E-07	1.109(1.066-1.154)	0.000848	1.101(1.041-1.166)	0.0000000838
rs4890010	17	A	1803	5725	4342	0.393	13498	44838	37158	0.3761	1.70E-07	1.078(1.048-1.109)	6.65E-07	1.107(1.064-1.153)	0.000895	1.096(1.038-1.158)	0.00000017

rs8080730	17	A	1770	5702	4370	0.3902	13295	44612	37433	0.3734	2.76E-07	1.077(1.047-1.108)	6.71E-07	1.107(1.064-1.153)	0.00168	1.092(1.034-1.153)	0.9173
rs4890012	17	C	1794	5725	4352	0.3923	13485	44844	37182	0.3759	3.62E-07	1.076(1.046-1.107)	1.16E-06	1.105(1.061-1.15)	0.001393	1.093(1.035-1.154)	LD
**Severe headache**																	
rs13272202	8	C	65	566	1231	0.1869	2170	24317	69416	0.1494	1.02E-09	1.299(1.195-1.413)	6.05E-09	1.337(1.213-1.475)	0.001203	1.523(1.181-1.964)	Top SNP
rs2195515	8	G	66	574	1230	0.1888	2238	24697	69407	0.1514	1.36E-09	1.295(1.191-1.407)	6.63E-09	1.334(1.211-1.471)	0.001708	1.498(1.164-1.928)	0.1401
rs10955583	8	T	66	574	1226	0.1892	2253	24812	69326	0.1521	1.76E-09	1.292(1.189-1.405)	8.34E-09	1.332(1.208-1.469)	0.001849	1.493(1.16-1.922)	0.00000000176
rs9297447	8	A	67	575	1228	0.1896	2279	24960	69346	0.1528	2.41E-09	1.289(1.186-1.401)	1.32E-08	1.326(1.203-1.462)	0.001498	1.501(1.168-1.928)	0.08363
rs11776383	8	T	66	576	1229	0.1892	2277	24936	69405	0.1526	2.87E-09	1.288(1.185-1.4)	1.21E-08	1.327(1.204-1.463)	0.002376	1.479(1.149-1.903)	0.195
rs13260231	8	A	66	576	1229	0.1892	2277	24936	69405	0.1526	2.87E-09	1.288(1.185-1.4)	1.21E-08	1.327(1.204-1.463)	0.002376	1.479(1.149-1.903)	0.195

rs1896861	8	C	66	576	1230	0.1891	2270	24928	69406	0.1525	2.89E-09	1.288(1.184-1.4)	1.23E-08	1.327(1.204-1.463)	0.002352	1.479(1.149-1.904)	0.16
rs10093394	8	T	66	568	1231	0.1877	2219	24702	69461	0.1512	3.02E-09	1.289(1.185-1.402)	1.94E-08	1.323(1.2-1.459)	0.001203	1.518(1.179-1.954)	0.7549
rs10108913	8	T	66	575	1229	0.189	2274	24927	69401	0.1526	3.2E-09	1.287(1.184-1.399)	1.41E-08	1.326(1.203-1.462)	0.002259	1.482(1.151-1.907)	0.195
rs5894044	8	A	66	575	1229	0.189	2274	24931	69403	0.1526	3.23E-09	1.287(1.184-1.399)	1.43E-08	1.326(1.203-1.461)	0.002255	1.482(1.151-1.907)	0.195
rs6469357	8	A	66	576	1230	0.1891	2277	24948	69405	0.1527	3.28E-09	1.286(1.183-1.398)	1.38E-08	1.326(1.203-1.461)	0.002456	1.477(1.148-1.901)	0.1625
rs12678206	8	T	66	576	1230	0.1891	2277	24951	69406	0.1527	3.31E-09	1.286(1.183-1.398)	1.39E-08	1.326(1.203-1.461)	0.002453	1.477(1.148-1.901)	0.1625
rs1160551	8	C	66	576	1230	0.1891	2277	24951	69404	0.1527	3.33E-09	1.286(1.183-1.398)	1.4E-08	1.326(1.203-1.461)	0.002454	1.477(1.148-1.901)	0.1625
rs12676588	8	G	66	576	1230	0.1891	2277	24951	69404	0.1527	3.33E-09	1.286(1.183-1.398)	1.4E-08	1.326(1.203-1.461)	0.002454	1.477(1.148-1.901)	0.1625
rs12682170	8	C	66	576	1230	0.1891	2277	24951	69404	0.1527	3.33E-09	1.286(1.183-1.398)	1.4E-08	1.326(1.203-1.461)	0.002454	1.477(1.148-1.901)	0.1625

rs1429439	8	T	66	576	1230	0.1891	2277	24951	69404	0.1527	3.33E-09	1.286(1.183-1.398)	1.4E-08	1.326(1.203-1.461)	0.002454	1.477(1.148-1.901)	0.1625
rs1429440	8	A	66	576	1230	0.1891	2277	24951	69404	0.1527	3.33E-09	1.286(1.183-1.398)	1.4E-08	1.326(1.203-1.461)	0.002454	1.477(1.148-1.901)	0.1625
rs1579468	8	G	66	576	1230	0.1891	2277	24951	69404	0.1527	3.33E-09	1.286(1.183-1.398)	1.4E-08	1.326(1.203-1.461)	0.002454	1.477(1.148-1.901)	0.1625
rs2195516	8	A	66	576	1230	0.1891	2277	24951	69404	0.1527	3.33E-09	1.286(1.183-1.398)	1.4E-08	1.326(1.203-1.461)	0.002454	1.477(1.148-1.901)	0.1625
rs959495	8	A	66	576	1230	0.1891	2277	24951	69404	0.1527	3.33E-09	1.286(1.183-1.398)	1.4E-08	1.326(1.203-1.461)	0.002454	1.477(1.148-1.901)	0.1625
rs4876681	8	C	66	576	1230	0.1891	2277	24952	69402	0.1527	3.35E-09	1.286(1.183-1.398)	1.41E-08	1.326(1.203-1.461)	0.002455	1.477(1.148-1.901)	0.1784
rs11365170	8	G	65	565	1232	0.1866	2188	24543	69479	0.1503	3.41E-09	1.289(1.185-1.402)	2.19E-08	1.323(1.199-1.459)	0.001245	1.521(1.179-1.961)	0.9299
rs10087862	8	C	66	572	1230	0.1884	2264	24884	69429	0.1523	4.21E-09	1.285(1.182-1.397)	1.99E-08	1.322(1.199-1.458)	0.002083	1.487(1.155-1.913)	0.00000000421
rs6469358	8	C	66	574	1231	0.1887	2270	24924	69414	0.1525	4.4E-09	1.284(1.181-1.396)	1.98E-08	1.322(1.199-1.457)	0.002286	1.481(1.151-1.906)	0.0000000044

rs1115957	8	A	66	572	1230	0.1884	2267	24906	69407	0.1524	4.84E-09	1.284(1.181-1.396)	2.28E-08	1.321(1.198-1.456)	0.002148	1.485(1.154-1.911)	0.00000000484
rs13260568	8	T	66	572	1230	0.1884	2267	24906	69407	0.1524	4.84E-09	1.284(1.181-1.396)	2.28E-08	1.321(1.198-1.456)	0.002148	1.485(1.154-1.911)	LD
rs4876697	8	T	66	572	1230	0.1884	2267	24906	69407	0.1524	4.84E-09	1.284(1.181-1.396)	2.28E-08	1.321(1.198-1.456)	0.002148	1.485(1.154-1.911)	0.00000000484
rs4876699	8	G	66	572	1230	0.1884	2267	24906	69407	0.1524	4.84E-09	1.284(1.181-1.396)	2.28E-08	1.321(1.198-1.456)	0.002148	1.485(1.154-1.911)	0.00000000484
chr8:111500183_AT_A	8	A	64	571	1222	0.1882	2231	24636	68959	0.1518	5.58E-09	1.284(1.18-1.396)	1.85E-08	1.324(1.201-1.46)	0.003986	1.457(1.128-1.882)	LD
rs6469359	8	A	66	572	1231	0.1883	2269	24915	69407	0.1525	5.65E-09	1.282(1.179-1.394)	2.68E-08	1.319(1.196-1.454)	0.002179	1.484(1.153-1.91)	0.00000000565
rs7000860	8	G	66	572	1231	0.1883	2269	24915	69406	0.1525	5.66E-09	1.282(1.179-1.394)	2.68E-08	1.319(1.196-1.454)	0.002179	1.484(1.153-1.91)	0.00000000566

Chr., chromosome; RAF, risk allele frequency. (a) Allele 1, risk allele; allele 2, non-risk allele. (b) P values and ORs were calculated by logistic regression analysis, with age, gender, and 10 principal components as covariates. Non-risk alleles were considered as references in the three genetic models: additive, 1 versus 2; recessive, 11 versus 12 + 22; dominant, 11 + 12 versus 22. Heterogeneity across the two stages was examined by the Cochran Q test under a genetic model which provided the minimum P value in the screening stage. (c) ORs and P values were calculated using the Mantel-Haenszel fixed-effects model.

Supplementary Table2. Functional annotation of associated variants and candidate gene mapping by FUMA

rsID	Chromosome	Position	non_effect_allele	effect_allele	Minor allele frequency	*P*	nearest Gene	Function
**Phenotype: Broadly-defined headache**								
rs8072917	17	78309108	C	T	0.3661	4.49E-08	RNF213	intronic
rs8078851	17	78326267	C	T	0.3651	5.54E-08	RNF213:CTD-2047H16.4	ncRNA_exonic
rs9674961	17	78319136	G	A	0.376	8.06E-08	RNF213	exonic
rs4890009	17	78311508	G	A	0.375	8.38E-08	RNF213	exonic
rs4890010	17	78316179	G	A	0.38	1.70E-07	RNF213	intronic
rs8080730	17	78316609	G	A	0.377	2.76E-07	RNF213	intronic
rs55692616	17	78322936	A	C	0.3452	3.23E-06	RNF213	intronic

rs56127781	17	78323311	C	T	0.3462	3.52E-06	RNF213	intronic
rs8067292	17	78333840	T	C	0.3423	3.84E-06	RNF213:CTD-2047H16.4	ncRNA_intronic
rs35993981	17	78323445	T	A	0.3462	3.98E-06	RNF213	intronic
rs9944443	17	78324048	T	A	0.3472	4.10E-06	RNF213	intronic
rs8078251	17	78334206	G	A	0.3353	4.40E-06	RNF213:CTD-2047H16.4	ncRNA_intronic
rs4890015	17	78335200	G	A	0.3353	4.52E-06	RNF213:CTD-2047H16.4	ncRNA_intronic
rs4890014	17	78335056	C	T	0.3343	4.57E-06	RNF213:CTD-2047H16.4	ncRNA_intronic
rs7211876	17	78311360	C	T	0.3214	5.14E-06	RNF213	intronic
rs60117041	17	78340931	GA	G	0.3849	9.43E-05	RNF213:CTD-2047H16.4	ncRNA_intronic

rs8070106	17	78344446	G	A	0.3849	0.0001085	RNF213:CTD-2047H16.4	ncRNA_intronic
**Phenotype: Severe headache**								
rs13272202	8	112545515	G	C	0.1438	1.02E-09	RP11-1101K5.1	ncRNA_intronic
rs2195515	8	112541791	C	G	0.1438	1.36E-09	RP11-1101K5.1	ncRNA_intronic
rs10955583	8	112508498	G	T	0.1438	1.76E-09	RP11-1101K5.1	ncRNA_intronic
rs9297447	8	112515260	G	A	0.1448	2.41E-09	RP11-1101K5.1	ncRNA_intronic
rs11776383	8	112524396	G	T	0.1438	2.87E-09	RP11-1101K5.1	ncRNA_intronic
rs13260231	8	112526408	T	A	0.1438	2.87E-09	RP11-1101K5.1	ncRNA_intronic
rs1896861	8	112537118	G	C	0.1448	2.89E-09	RP11-1101K5.1	ncRNA_intronic
rs10093394	8	112548592	C	T	0.1438	3.02E-09	RP11-1101K5.1	ncRNA_intronic

rs10108913	8	112514927	C	T	0.1438	3.20E-09	RP11-1101K5.1	ncRNA_intronic
rs5894044	8	112516046	AT	A	0.1438	3.23E-09	RP11-1101K5.1	ncRNA_intronic
rs6469357	8	112545052	G	A	0.1438	3.28E-09	RP11-1101K5.1	ncRNA_intronic
rs12678206	8	112528267	C	T	0.1438	3.31E-09	RP11-1101K5.1	ncRNA_intronic

Supplementary Table 3. Top 10 gene sets results by MAGMA integrated in FUMA for the phenotype of headache (A) and severe headache (B)

A

Number of Genes	Beta	Standard error	*P*	Full names of gene sets
6	1.1551	0.2961	4.81E-05	GO_cc:go_picln_sm_protein_complex
25	0.6824	0.1776	6.12E-05	Curated_gene_sets:bandres_response_to_carmustin_without_mgmt_48hr_dn
199	0.21539	0.057363	8.71E-05	GO_cc:go_condensed_chromosome
89	0.30895	0.083467	0.00010755	GO_cc:go_condensed_nuclear_chromosome
118	0.29205	0.080699	0.00014834	Curated_gene_sets:johnstone_parvb_targets_2_up
194	0.21637	0.060951	0.00019332	Curated_gene_sets:benporath_myc_targets_with_ebox
160	0.22274	0.063815	0.00024188	Curated_gene_sets:fernandez_bound_by_myc
18	0.67989	0.19516	0.00024783	Curated_gene_sets:biocarta_nos1_pathway
5	1.0577	0.30742	0.00029097	GO_bp:go_regulation_of_cytoplasmic_translational_initiation
10	1.0161	0.29772	0.00032235	GO_cc:go_cell_trailing_edge

B

Number of Genes	Beta	Standard error	*P*	Full names of gene sets
124	0.32157	0.078635	2.17E-05	GO_bp:go_endothelial_cell_proliferation
19	0.75928	0.18608	2.26E-05	Curated_gene_sets:reactome_signal_transduction_by_l1
83	0.38109	0.094429	2.73E-05	GO_bp:go_positive_regulation_of_endothelial_cell_proliferation
10	1.0949	0.27172	2.81E-05	GO_bp:go_positive_regulation_of_methylation_dependent_chromatin_silencing
98	0.33108	0.085532	5.45E-05	GO_bp:go_blood_vessel_endothelial_cell_migration

25	0.6775	0.17785	6.99E-05	Curated_gene_sets:amit_serum_response_40_mcf10a
4	1.2153	0.32846	0.000108	GO_cc:go_eukaryotic_translation_initiation_factor_2_complex
91	0.3444	0.094373	0.000132	GO_bp:go_positive_regulation_of_endothelial_cell_migration
5	1.4876	0.42071	0.000204	GO_bp:go_branched_chain_amino_acid_transport
45	0.45923	0.1299	0.000204	Curated_gene_sets:pid_angiopoietin_receptor_pathway

Supplementary Table 4. Tissue expression analysis on 53 specific tissue types for broadly-defined headache (A) and severe headache, and tissue expression analysis on 30 general tissue types for broadly-defined headache (C) and severe headache (D).

A. Broadly-defined headache, 53 specific tissue types

Tissues	Beta	Standard error	P
Pancreas	0.017049	0.008919	0.027975
Artery_Tibial	0.008406	0.01062	0.21431
Heart_Atrial_Appendage	0.007495	0.010164	0.23045
Prostate	0.00855	0.012893	0.25362
Brain_Hypothalamus	0.005686	0.008587	0.25394
Artery_Aorta	0.00695	0.010845	0.26081
Bladder	0.007842	0.012305	0.26197
Artery_Coronary	0.007797	0.012544	0.26711
Lung	0.006046	0.00987	0.2701
Brain_Amygdala	0.004528	0.008382	0.29453
Heart_Left_Ventricle	0.004635	0.009521	0.31319
Brain_Anterior_cingulate_cortex_BA24	0.003592	0.007882	0.32429
Spleen	0.003249	0.00774	0.33735
Brain_Frontal_Cortex_BA9	0.002624	0.007283	0.35931
Cervix_Ectocervix	0.004339	0.013594	0.37479
Vagina	0.003731	0.011745	0.37538

Brain_Cortex	0.0022	0.007607	0.38622
Brain_Hippocampus	0.002447	0.008566	0.38758
Esophagus_Gastroesophageal_Junction	0.0038	0.013468	0.38892
Nerve_Tibial	0.002596	0.010848	0.40545
Brain_Spinal_cord_cervical_c-1	0.001912	0.009202	0.4177
Brain_Nucleus_accumbens_basal_ganglia	0.00161	0.008096	0.42119
Cells_Transformed_fibroblasts	0.001422	0.007194	0.42166
Brain_Caudate_basal_ganglia	0.001563	0.008499	0.42707
Brain_Substantia_nigra	0.001571	0.009122	0.43165
Brain_Cerebellum	0.001148	0.006715	0.43211
Brain_Putamen_basal_ganglia	0.001195	0.008445	0.44374
Colon_Sigmoid	0.001755	0.01293	0.44601
Colon_Transverse	0.00113	0.011774	0.46178
Whole_Blood	0.00053	0.006024	0.46492
Pituitary	0.000713	0.009614	0.47044
Brain_Cerebellar_Hemisphere	0.000382	0.006481	0.47651
Breast_Mammary_Tissue	0.000374	0.01338	0.48885
Uterus	0.000219	0.011596	0.49247

Esophagus_Muscularis	-0.00014	0.013046	0.50431
Adipose_Subcutaneous	-0.00126	0.011417	0.54407
Minor_Salivary_Gland	-0.00122	0.010389	0.54663
Small_Intestine_Terminal_Ileum	-0.00128	0.009776	0.55215
Cervix_Endocervix	-0.0024	0.012097	0.57865
Esophagus_Mucosa	-0.00277	0.008126	0.63334
Ovary	-0.00385	0.010155	0.64787
Skin_Sun_Exposed_Lower_leg	-0.00422	0.008348	0.6933
Stomach	-0.0065	0.012573	0.69748
Skin_Not_Sun_Exposed_Suprapubic	-0.00492	0.0084	0.72105
Muscle_Skeletal	-0.00445	0.007401	0.72635
Liver	-0.00517	0.006696	0.77981
Adipose_Visceral_Omentum	-0.00975	0.011903	0.79362
Kidney_Cortex	-0.00854	0.009714	0.81042
Fallopian_Tube	-0.01117	0.012474	0.81468
Adrenal_Gland	-0.01134	0.010506	0.8597
Thyroid	-0.0116	0.010314	0.86972
Cells_EBV-transformed_lymphocytes	-0.00613	0.005215	0.88008

Testis	-0.00724	0.006039	0.88458

B. Severe headache, 53 specific tissue types

Tissues	Beta	Standard error	P
Uterus	0.031668	0.011471	0.002888
Esophagus_Gastroesophageal_Junction	0.029358	0.013324	0.013793
Colon_Sigmoid	0.024833	0.012792	0.026123
Ovary	0.019291	0.010048	0.027442
Cervix_Endocervix	0.02261	0.011969	0.029449
Fallopian_Tube	0.022084	0.012342	0.036792
Nerve_Tibial	0.019105	0.010733	0.037543
Esophagus_Muscularis	0.022055	0.012908	0.043768
Artery_Tibial	0.014977	0.010508	0.077037
Artery_Aorta	0.015231	0.010731	0.077902
Thyroid	0.013003	0.010206	0.10133
Brain_Cerebellum	0.007255	0.006644	0.13745
Bladder	0.012371	0.012175	0.1548
Cervix_Ectocervix	0.012962	0.013451	0.16762
Brain_Cerebellar_Hemisphere	0.006171	0.006413	0.16796
Artery_Coronary	0.009925	0.012412	0.21197

Adipose_Subcutaneous	0.008549	0.011297	0.22461
Breast_Mammary_Tissue	0.0098	0.01324	0.22959
Adipose_Visceral_Omentum	0.008116	0.011778	0.24539
Pituitary	0.006279	0.009513	0.25461
Pancreas	0.003993	0.008826	0.3255
Heart_Atrial_Appendage	0.002376	0.010057	0.40663
Vagina	0.001665	0.011622	0.44305
Testis	0.000261	0.005976	0.48258
Heart_Left_Ventricle	0.000321	0.009422	0.48641
Colon_Transverse	-0.00084	0.01165	0.52887
Stomach	-0.00113	0.012442	0.53606
Prostate	-0.00244	0.012758	0.57579
Skin_Not_Sun_Exposed_Suprapubic	-0.00164	0.008312	0.57813
Brain_Frontal_Cortex_BA9	-0.00142	0.007206	0.57836
Brain_Cortex	-0.00156	0.007527	0.58227
Brain_Spinal_cord_cervical_c-1	-0.00225	0.009105	0.59763
Skin_Sun_Exposed_Lower_leg	-0.0026	0.008261	0.6234
Liver	-0.00266	0.006626	0.65622

Brain_Anterior_cingulate_cortex_BA24	-0.00364	0.0078	0.67978
Brain_Hippocampus	-0.00464	0.008476	0.70779
Kidney_Cortex	-0.00541	0.009612	0.71339
Brain_Nucleus_accumbens_basal_ganglia	-0.00519	0.008011	0.74164
Brain_Caudate_basal_ganglia	-0.00602	0.00841	0.76297
Muscle_Skeletal	-0.00525	0.007323	0.76332
Brain_Amygdala	-0.00602	0.008294	0.76597
Small_Intestine_Terminal_Ileum	-0.00705	0.009673	0.76704
Brain_Putamen_basal_ganglia	-0.00646	0.008356	0.78041
Esophagus_Mucosa	-0.00646	0.008041	0.78916
Lung	-0.00841	0.009766	0.80546
Brain_Substantia_nigra	-0.00833	0.009026	0.82187
Spleen	-0.00713	0.007659	0.82416
Cells_EBV-transformed_lymphocytes	-0.00528	0.00516	0.8471
Minor_Salivary_Gland	-0.01086	0.010279	0.85468
Brain_Hypothalamus	-0.0115	0.008496	0.91203
Cells_Transformed_fibroblasts	-0.01191	0.007118	0.9529
Adrenal_Gland	-0.01813	0.010396	0.95937

Whole_Blood	-0.01471	0.00596	0.99322

c. Broadly-defined headache, 30 general tissue types

Tissues	Beta	Standard error	P
Pancreas	0.019615	0.009385	0.018321
Blood_Vessel	0.012036	0.012444	0.16673
Prostate	0.013883	0.01499	0.1772
Bladder	0.013406	0.014708	0.18103
Lung	0.009188	0.011451	0.21118
Heart	0.007634	0.010135	0.22567
Spleen	0.004336	0.008325	0.30123
Vagina	0.006833	0.013583	0.30746
Brain	0.00335	0.007321	0.32363
Nerve	0.004421	0.011174	0.34618
Colon	0.005788	0.016614	0.36377
Cervix_Uteri	0.004328	0.016214	0.39476

Pituitary	0.001947	0.008905	0.41348
Breast	0.003369	0.016751	0.42029
Uterus	0.002233	0.012736	0.43043
Esophagus	-6.36E-05	0.018119	0.5014
Salivary_Gland	-0.0004	0.011867	0.51359
Small_Intestine	-0.0007	0.011018	0.52541
Blood	-0.00159	0.006732	0.5936
Ovary	-0.00295	0.010796	0.60749
Skin	-0.00347	0.011135	0.62228
Adipose_Tissue	-0.00505	0.01413	0.63973
Stomach	-0.00654	0.014441	0.67478
Muscle	-0.00374	0.007387	0.69385
Liver	-0.00527	0.00697	0.77534
Kidney	-0.00884	0.010409	0.80224
Fallopian_Tube	-0.01317	0.014628	0.81605

Adrenal_Gland	-0.01068	0.010879	0.83689
Testis	-0.00696	0.006052	0.8748
Thyroid	-0.01345	0.011639	0.87612

D. Severe headache, 30 general tissue types

Tissues	Beta	Standard error	P
Uterus	0.036455	0.012599	0.001908
Fallopian_Tube	0.027983	0.014474	0.026605
Ovary	0.020587	0.010681	0.026971
Nerve	0.019091	0.011055	0.042109
Cervix_Uteri	0.025632	0.016042	0.05506
Blood_Vessel	0.01677	0.012313	0.086614
Thyroid	0.014987	0.011517	0.096593
Bladder	0.015156	0.014553	0.14885
Colon	0.015237	0.01644	0.17701
Breast	0.012089	0.016575	0.23289

Adipose_Tissue	0.010085	0.013981	0.23535
Esophagus	0.012008	0.017929	0.2515
Pituitary	0.005021	0.008811	0.28439
Pancreas	0.003607	0.009288	0.34888
Heart	0.000627	0.010028	0.47509
Vagina	0.00015	0.013441	0.49556
Testis	5.45E-05	0.005989	0.49637
Brain	-0.00132	0.007244	0.57246
Stomach	-0.00367	0.01429	0.60146
Prostate	-0.00572	0.014834	0.65007
Liver	-0.00338	0.006897	0.68779
Kidney	-0.00728	0.0103	0.76019
Muscle	-0.00564	0.007309	0.77981
Small_Intestine	-0.01035	0.010902	0.82887
Spleen	-0.00906	0.008237	0.86442

Skin	-0.01218	0.011018	0.86556
Lung	-0.01296	0.011331	0.87356
Salivary_Gland	-0.0158	0.011741	0.91076
Adrenal_Gland	-0.02054	0.010764	0.97179
Blood	-0.01644	0.00666	0.99322

Supplementary Table 5. Pain-related questionnaire in Taiwan biobank.

E-2-1. I will describe some physical pains below. Could you please tell me if you have had pain in this area in the past three months?

[If you had pain, please tell us: Is it often painful or only occasionally painful?]

[Please note: If you had headache or migraine, please keep answering E-2-4-1~E-2-4-4, and for women who have not menopause, please keep answering 6 dysmenorrhea.]

<table>
<tr><td rowspan="2">Physical pain</td><td rowspan="2">Continuing answering the questions to the right if you checked this item</td><td colspan="3">Frequency of pain</td></tr>
<tr><td>Constant</td><td>Come and go</td><td>Prefer not to say</td></tr>
<tr><td>1. Joint pain and stiffness all over the body</td><td>-></td><td>1</td><td>2</td><td>77</td></tr>
<tr><td>2. Neck or shoulder pain</td><td>-></td><td>1</td><td>2</td><td>77</td></tr>
<tr><td>3. Low back pain</td><td>-></td><td>1</td><td>2</td><td>77</td></tr>
<tr><td>4. Sciatic nerve pain</td><td>-></td><td>1</td><td>2</td><td>77</td></tr>
<tr><td>5. Headache, migraine</td><td>-></td><td>1</td><td>2</td><td>77</td></tr>
<tr><td colspan="5">E-2-4-1. Has a headache affected your work, study, or daily life?
1. Yes
2. No</td></tr>
</table>

E-2-4-2. What was the severity of your headache?

1. Mild
2. Moderate
3. Severe

E-2-4-3. Have you ever had nausea or vomiting when you had a headache?

1. Yes
2. No

E-2-4-4. Did you experience light sensitivity when you had a headache?

1. Yes
2. No

6. Dysmenorrhea	->	1	2	77
7. Other (If you had any diseases or symptoms, describe them)<br><br>Other (1)	->	1	2	77
Other (2)	->	1	2	77
Other (3)	->	1	2	77
Other (4)	->	1	2	77
